# Heat-Induced Changes in Avian (Cortical and Medullary) Bone Mineral Reactivity, Solubility, and Adsorption Capacity

**DOI:** 10.1021/acsomega.5c12924

**Published:** 2026-05-20

**Authors:** Tamara Pozo-Gualda, Monica Jimenez-Carretero, Pablo A. Rodriguez-Jimenez, Miguel Burgos-Ruiz, Concepcion Jimenez-Lopez, Alejandro B. Rodriguez-Navarro

**Affiliations:** † Depto Microbiologia, Facultad de Ciencias, Universidad de Granada, Granada 18071, Spain; ‡ Depto Mineralogia y Petrologia, Facultad de Ciencias, 16741 Universidad de Granada, Granada 18071, Spain

## Abstract

Two types of bone tissue from laying hens—cortical and medullary bone—were selected to investigate how differences in structure, organization, organic matrix composition, mineral properties, and functionality, further potentiated following upon a thermal treatment (up to 800 °C), influence mineral reactivity, dissolution, and adsorption behavior, using Pb as a model adsorbate. At lower temperatures (<400 °C), water and organic components are progressively removed, resulting in a more porous mineral structure with increased surface area. At higher temperatures (>600 °C), sintering promotes the coarsening of apatite crystals, leading to a reduction in surface area. Simultaneously, the loss of organic matter and the diffusion of foreign ions (e.g., Mg and Na) from the apatite lattice to the crystal surface alter both surface chemistry and charge. Bone mineral solubility and adsorption were strongly influenced by both bone type and treatment temperature. Medullary bone exhibited significantly higher solubility than cortical bone, particularly at intermediate treatment temperatures. This behavior appears to be primarily controlled by the organic matrix (e.g., collagen and proteoglycans) and the manner in which these components interact with or adsorb onto crystal surfaces. Pb adsorption was governed by both surface area and chemical composition, and predominantly occurred via a coupled dissolution–precipitation mechanism. Specifically, the dissolution of Ca-apatite was accompanied by the formation of highly insoluble Pb-bearing mineral phases at the bone surface. This process was notably more efficient in thermally treated medullary bone. Overall, this study demonstrates how key bone properties can be tailored for specific applications, highlighting the potential for revalorizing bone waste from the meat industry.

## Graphical abstract

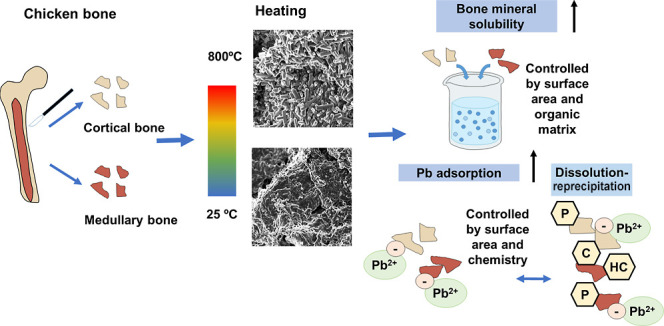

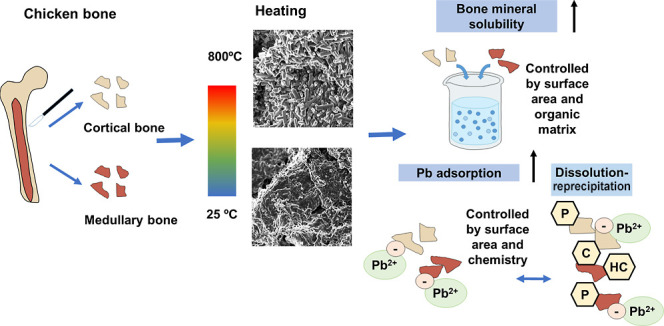

## 1 Introduction

The rapid expansion of global meat production, largely driven by the poultry industry, generates enormous amounts of waste that requires adequate and sustainable management strategies. In chickens, bones account for more than 10% of total body weight that needs to be disposed by incineration to minimize biological risks.^1^ However, this waste also represents a potential source of renewable energy and valuable byproducts.^1^

Bone is a composite material with a complex chemical composition and hierarchical structure, which serves different functions such as structural support and supply of ions (calcium, magnesium, and phosphorus) needed for basic cell functions.^2^ It consists primarily of an inorganic phase (nanocrystalline carbonated apatite; 60%), mineralizing an organic matrix (largely type I collagen; 30%), water (10%), and cells. Bone composition and architecture vary depending on their functionalities and locations in the skeleton. Bird bones have a thin outer cortex (made by dense cortical bone) that is reinforced internally by spongy or trabecular bone (Figure S1). Additionally, during reproduction (e.g., egg laying), female birds develop a special new type of bone tissue (medullary bone), which partially fills the marrow cavity of long bones and serves as a labile source of calcium for the rapid eggshell calcification.^3,4^ Unlike cortical and trabecular bones, medullary bone is formed by an organic matrix rich in proteoglycans and glycoproteins rather than collagen. This is a unique and relatively understudied bone tissue with an organic matrix that differs substantially from that of cortical or trabecular bone. The comparison of cortical and medullary bones provides a valuable opportunity to better understand the influence of organic components on bone mineral properties and their behavior following the removal of organic material during heating.

Apatite, from geological/synthetic/biological (bone-derived materials, i.e., bone char) origin is widely used for the remediation of heavy metal contamination in soils and water due to its capacity to incorporate foreign ions.^5–7^ Biological apatite (from bone) is a highly reactive material due to its high-surface area, solubility, high degree of ionic substitutions, and the presence of functional groups from organics.^8,9^ All these characteristics allow bone apatite to have a higher adsorption capacity than that of geological or synthetic apatite.^6,7,10–12^ Bone characteristics that determine mineral reactivity can greatly vary depending on the bone source (fish, bird, mammal), the type of bone (cortical, trabecular, medullar), and how the bone sample is processed for the specific application.^7,9,13–15^

Previous work from our group^16^ studied in detail the different evolution during heating (up to 800 °C) of bone chemistry, microstructure, and mineral crystallinity in cortical and medullary bone. In this study, we extend on these results to investigate how heat-induced changes in these two types of bone tissue influence their mineral reactivity and specifically its dissolution and adsorption behavior. The different organic matrix composition of these bone tissues provides a valuable opportunity to better understand the influence of organic components on bone mineral properties and behavior, as well as how these properties change following their removal. Batch experiments were conducted using thermally treated bone samples exposed to different solutions (acidic or Pb-bearing solutions) to study mineral dissolution, or Pb adsorption capacity of the different bone-derived materials. A combination of complementary analytical techniques such as electron microscopy (SEM, TEM), nitrogen adsorption isotherm (BET), zeta-potential, thermogravimetric analysis (TGA), X-ray diffraction (XRD), infrared (FTIR), and optical emission (ICP-OES) were used to study the chemical and structural characteristics of bone samples, as well as their dissolution and adsorption behavior. The results obtained in this study contribute to a deeper understanding of bone mineral dissolution and reactivity dynamics, as well as highlight the potential of bone waste-derived materials as a valuable residue for heavy metal contamination remediation.

## 2 Materials and Methods

### 2.1 Bone Samples and Thermal Treatment

Tibia bone samples (10) from white laying hens (Lohmann, Germany) at peak-lay (25 weeks old) were selected from a previous study (stored at −20 °C). One section (approximately 2–3 cm long) was cut from the middle shaft of each tibia. The cortical and medullary bone tissues were then mechanically separated using a scalpel. The cortical, or medullary tissues, from different tibias were combined and crushed with a hand hammer (into few mm pieces). The combined samples were split into 5 portions, and each portion was annealed at different temperatures (25 °C or untreated, 200 °C, 400 °C, 600 °C, and 800 °C) for 1 h in a Carbolite oven (model 1100). These temperatures were chosen from TGA-DSC analyses of bone samples and represent the temperatures at which main weight loss events occur during heating: due to water evaporation (25–200 °C), organic matter combustion (200–600 °C), and carbonate thermal decomposition and release of CO_2_ (600–800 °C) (see Supporting Information; Figure S2). For clarity, cortical bone samples are hereafter referred to as CB and medullary bone samples as MB, followed by the treatment temperature (i.e., CB25 °C, MB25 °C, etc.).

### 2.2 Bone Mineral Dissolution Experiments

Small pieces (2 × 2 mm) of cortical and medullary bone, treated at different temperatures, were immersed in 1 mL of 1% HCl acid solution for varying durations: 0, 5, 10, 20 min. After this treatment, bone samples were collected, rinsed twice with distilled water to remove any residual HCl acid, and dried at room temperature for 24 h. To determine the mineral dissolution kinetics, the calcium (and other ions) released into the HCl acid solution during bone mineral dissolution were analyzed by optical emission spectroscopy (ICP-OES) using a PERKIN-ELMER OPTIMA 8300 system. Prior to analysis, the pH was recorded, and the solutions filtered to remove any residual bone fragments using a 13 mm diameter, 0.45 μm pore size PES membrane filter.

### 2.3 Pb Adsorption on Thermally Treated Bones

A set of experiments were prepared to study Pb cation adsorption kinetics on bone samples treated at different temperatures. Bone samples (0.08 ± 0.01 g) were added in wells (24 well plates) containing 2.7 mL of Pb­(NO_3_)_2_ solutions with 25, 50, 75, 150, and 250 ppm of Pb and left for 1, 3, and 7 h. After the predefined times, Pb remaining in the solution was measured by ICP-OES. To complete this procedure, the solution was withdrawn and filtered using a 0.45 μm pore size PES membrane filter to remove any remaining bone debris. The amount of Pb adsorbed on bone samples was indirectly determined as following formula: retention percentage (%) = $\frac{\left(X_{0}-X_{n}\right)}{X_{0}}$ × 100.

The Pb adsorbed in the bone was calculated as the initial concentration of Pb in the solution (*X*_0_) minus the concentration of Pb at each time (*X*_*n*_). To account for experimental errors and for potential Pb adsorption on other surfaces, control experiments (without bone) at each Pb concentration were done.

Once the time to reach adsorption equilibrium was determined [occurring within the first few hours of reaction (<7 h)], Pb adsorption isotherms were performed. To this end, another set of batch experiments were run in parallel identically as detailed above; however, the reaction time was fixed and Pb­(NO_3_)_2_ solutions with increasing Pb concentration were used. In this case, to obtain sufficient experimental data points for improved model fitting (detailed below), adsorption experiments were conducted using Pb­(NO_3_)_2_ solutions with Pb concentrations up to 1450 ppm Pb (for untreated bones and bones treated at 400 °C) and up to 4500 ppm Pb (for bones treated at 600 and 800 °C). The pH of Pb­(NO_3_)_2_ solutions containing Pb concentrations (25 ppm, 1450 ppm, 4500 ppm) was measured over a 48 h period. At the specified time intervals, the solutions were withdrawn and filtered and the concentration of Pb was determined by ICP-OES, as indicated above. The amount of adsorbed Pb per unit mass of the bone sample at each Pb concentration in solution [*Q*_e_ (mg Pb/g bone)] was determined for each experiment as the difference between X_0_ and X_n_, normalized to the grams of bone used. To represent the adsorption isotherms of Pb for each type of bone, *Q*_e_ was plotted as a function of *C*_e_ (mg/L), which is the amount of nonadsorbed Pb at equilibrium. The resulting curves were fitted to the Freundlich, Langmuir, and Langmuir–Freundlich models defined by the eqs 1, 2, and 3, respectively.

1 $$Q_{e}=K_{F}C_{e}^{1/n}$$

2 $$Q_{e}=Q_{\max}\frac{C_{e}K_{L}}{1+C_{e}K_{L}}$$

and

3 $$Q_{e}=Q_{\max}\frac{{\left(K_{LF}C_{e}\right)}^{r}}{1+{\left(K_{LF}C_{e}\right)}^{r}}$$

in these equations, *Q*_max_ (mg Pb/g bone) is the maximum Pb adsorption capacity of the bone sample; *K*_F_ (mg Pb ^1–1/*n*^ L^1/*n*^/g bone), *K*_L_ (L/mg), and *K*_LF_ (L/mg) are the affinity constants for the Freundlich, Langmuir, and Langmuir–Freundlich models, respectively, 1/*n* is adsorption intensity, and *r* is the cooperativity coefficient.^17,18^

### 2.4 Characterization

#### 2.4.1 Electron Microscopy

Bone samples were coated with carbon using an EMITECH K975X evaporator prior to observation by scanning electron microscopy (SEM) (Auriga, Carl Zeiss; or Thermo Fisher Phenom XL G2). For transmission electron microscopy (TEM), grounded bone samples were collected on TEM copper grids and analyzed using a Thermo Fisher TALOS F200X TEM equipped with EDX detectors. To visualize the presence of Pb in bone, MB600 °C samples incubated in Pb­(NO_3_)_2_ were analyzed using high-resolution TEM (HRTEM, FEI TITAN G2) in the scanning transmitted electron (STEM) imaging mode.

#### 2.4.2 X-ray Diffraction

The mineralogy and microstructure of bone samples were analyzed using a powder X-ray diffractometer Bruker D8 DISCOVER with detector PILATUS3R 100 K-A, operating in the reflection mode using copper radiation to determine Theta-2Theta scans (from 4° to 85° with 0.02° step size in 2θ and 40 s integration time per step). Identification of mineral phases and analyses of XRD profiles were done using TOPAS 5.0 software (Bruker, Karlsruhe, Germany). The Rietveld method was used to refine XRD data and determine changes in apatite mineral during heating, dissolution, or Pb absorption experiments. The variables refined were the apatite unit cell parameters (a and c) and peak widths to determine crystallite sizes.

#### 2.4.3 BET Surface Area and Pore Size

Nitrogen adsorption–desorption isotherms were determined at a temperature of 77 K using the Micromeritics TriStar 3000 instrument (Figure S3). The samples were first degassed at 100 °C and 0.2 atm overnight using a Flowprep from Micromeritics. The Brunauer–Emmett–Teller (BET) model was then applied to determine the specific surface area (SSA), porosity volume, area, and size using the Barret–Joyner–Halenda (BJH) model, which was obtained from Micromeritics software.

#### 2.4.4 Fourier-Transform Infrared and Thermogravimetry

FT-IR was used to study changes in the surface chemistry of cortical and medullary bone samples following Pb adsorption. Bone samples (dried and grounded) were measured with an FTIR spectrometer (model 6600, JASCO, Japan) equipped with an ATR ProOne system. The infrared spectra were acquired in the absorbance mode over the range of 400 to 4000 cm^–1^, with a resolution of 2 cm^–1^ and 100 scans.

The relative amounts of water, organic matter, phosphate, and carbonate in representative bone samples (cortical and medullary bone) were determined by thermogravimetric analysis using approximately 30 mg in powder form and a TGA system from METTLER-TOLEDO (model TGA/DSC1). A heating rate of 20 °C/min in air was used for registering the TGA curves.

#### 2.4.5 Zeta Potential

Surface charge of bone mineral in the pH range at which the experiments were performed was measured in untreated and treated bones at 25 °C in a Zetasizer Nano-ZS (Malvern Instruments, Malvern, UK). Bone particles were suspended in 10 mL flasks of 10 mM NaClO_4_ 10 mM at pH 5 to 11 and sonicated for 2 min before each measurement. This pH range was selected to include that in which adsorption experiments were performed (Table S1).

### 2.5 Statistical Analyses

All results are presented as the mean ± standard deviation from three replicates. Comparisons between 2 groups were performed using an unpaired two-tailed *t*-Test via GraphPad Prism software with a significance level of 0.05 (α = 0.05). The “*p*” values were considered statistically significant when they were less than 0.05 (*), 0.01 (**), or 0.001 (***). The fits of the adsorption isotherm models were performed using OriginPro 2024 software.

## 3 Results and Discussion

### 3.1 Effect of Heating on Bone Microstructure, Porosity, Surface Area, and Surface Charge

#### 3.1.1 Bone Microstructure

Untreated chicken bone (tibia) has a dense outer cortex (cortical bone; about 0.5 mm thick) surrounding the medullar cavity, which is partially filled with medullary bone in female birds during the reproductive phase (egg-laying period) (Figure S1A).^3^ Cortical bone is organized into cylindrical structural units, or osteons, with osteocytes distributed and arranged concentrically around a central (Haversian) that house blood vessels and nerves (Figure S1B). At the microscale level, this tissue is composed of bundles of densely packed collagen fibrils mineralized by apatite nanocrystals. Apatite nanocrystals (about 20 × 5 nm in size) are elongated along their *c*-axis and are preferentially oriented parallel to the collagen fibrils. In contrast, the medullary bone consists of small, isolated trabeculae elements and functions as a calcium reservoir for the eggshell formation. Its organic matrix is rich in proteoglycans and glycoproteins and has a low collagen content.^19^ It is mineralized by small aggregates of randomly oriented apatite nanocrystals and foci. Apatite crystals in medullary bone are likewise elongated along the *c*-axis but have a smaller size (about 15 nm × 2 nm) than those of cortical bone.

The microstructural evolution of cortical and medullary bone upon thermal treatments was studied using SEM (Figure 1A–D for cortical bone, and Figure 1E–H for medullary bone). The surface of untreated cortical bone (CB25 °C) is quite smooth due to a high amount of organic matter (about 30% by weight) that completely covers the mineral component (Figure 1A). After annealing at 400 °C for 1 h, cortical bone has lost most of the organic components (only 5% by weight remain; determined by TGA). This loss generates a high microporosity and exposes the mineral phase, which consists of apatite nanoparticles arranged in strings, that remains partially covered with the residual organic matter (Figure 1B2). These string-like assemblies originate from the mineralized collagen fibrils that form the cortical bone tissue. Once the collagen is thermally degraded and lost, the remaining mineral phase persists. The apatite nanoparticles retain the original orientation that had in the collagen fibers but acquire a granular shape (about 20 nm in size; Figure 1B2). In bone samples treated at 600 °C, CB600 °C, all organic matter has been removed, resulting in a notable increase in microporosity (Figure 1C). The remaining mineral phase consists of strings of aligned granular apatite nanoparticles that have acquired a larger size (up to 60 nm) during sintering. In bone samples treated at 800 °C, CB800 °C, the apatite particles have further increased in size, forming aggregates of rounded crystals approximately 60–80 nm (Figure 1D2). At this temperature, the original mineral organization has been partially lost and the crystals are more randomly oriented.

**Figure 1:**
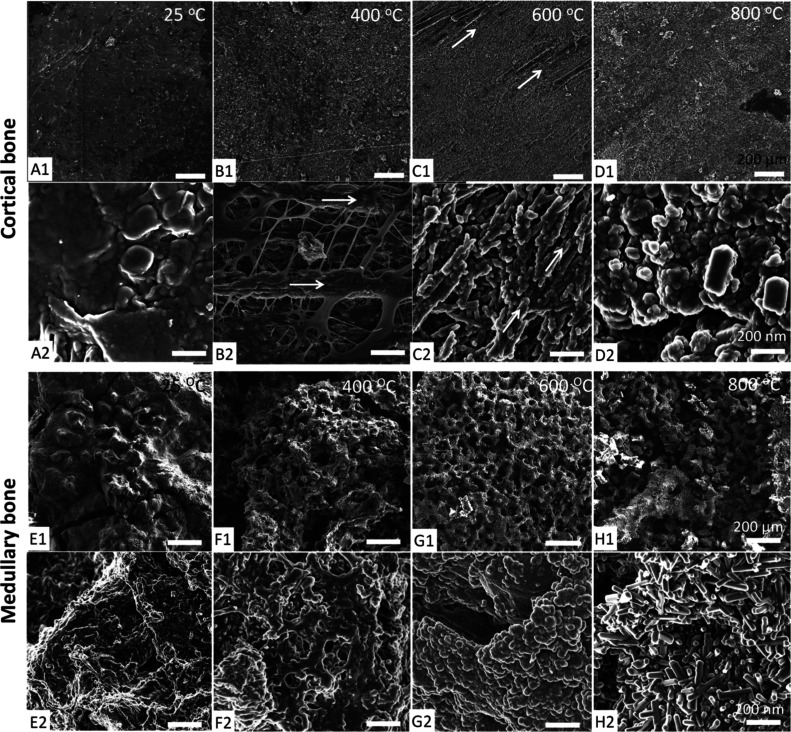
Electron microscopy of chicken bone at the micro- and nano scale. SEM images of cortical bone treated at (A) 25 °C (untreated); (B) 400 °C; (C) 600 °C; (D) 800 °C. There is an increase in porosity and apatite crystal size during heating (only appreciable at high magnification), while the organization of the mineral in parallel arrays of mineralized collagen fibrils is preserved (arrows). SEM images of medullary bone treated at (E) 25 °C (untreated); (F) 400 °C; (G) 600 °C; (H) 800 °C: medullary bone develop during heating a highly porous mineral structure resembling a coralline mineral. There is also a notable coarsening of apatite crystals at higher temperatures (>600 °C). Scale bars: 1:200 μm; 2:200 nm.

Medullary bone samples treated at different temperatures exhibited more pronounced changes than cortical bone samples (Figure 1E–H). Untreated medullary bone (MB25 °C) consists of isolated mineral trabecula (about 30–35% by weight) protruding from a mass of organic matter (about 65–60% by weight) in which they are embedded (Figure 1E). After heating to 400 °C (MB400 °C), most of the organic matter is lost, leaving a highly porous mineral structure that resembles a coralline mineral (i.e., Porites sp; Figure 1F). Pores of approximately 50 μm in diameter are evenly spaced and separated by 20 μm thick mineral walls (Figure 1F1). The walls are made from the mineralized trabeculae elements. Some remnants of the organic matter coating the mineral are still visible, and the crystals are slightly larger than those of the untreated bone (Figure 1F2). After heating at 600 °C (MB600 °C), all organic material has been lost, leaving only the mineral phase (Figure 1G). Apatite nanocrystals have fused together forming larger granular particles of approximately 100–200 nm. After heating at 800 °C (MB800 °C), the porous mineral structure has partially collapsed and mineral particles are now loose (Figure 1H). The apatite crystals have grown significantly in size (up to 100 nm in length) and are bound by well-defined prismatic faces (Figure 1H2). The observed increase in the size of apatite nanocrystals, in both cortical and medullary bone during heating, can also be observed by XRD analysis (Figure S4), which provides crystallite size values comparable to those observed by SEM. XRD data show that in cortical bone, apatite crystallite size remains nearly constant (around 18 × 5 nm) until 600 °C, then gradually increases with treatment temperature (up to 70 nm at 800 °C). In the case of medullary bone, the crystallite size remains nearly constant (around 8–9 nm) until 400 °C and starts to increase above this temperature (up to 100 nm at 800 °C). These results are consistent with previous studies.^15,16^

Upon heating at higher temperatures (>600 °C), the increase in apatite crystal size during annealing could result from solid-state mass transport from smaller to larger crystals, and/or from the fusion or coalescence of closely packed crystals into larger ones (sintering).^16,20,21^ In bone mineral, noncollagenous proteins (NCPs), phosphoproteins (i.e., osteocalcin and osteopontin) are strongly bound to apatite crystal surfaces, regulating apatite nucleation and growth.^22,23^ It is not until all organic components coating apatite crystals are completely removed that crystals start growing in all directions, developing rounded or equiaxial morphologies.^15,16,24^ Although collagen (the main organic component of cortical bone), and NCPs decompose at lower temperatures (around 400 °C) than proteoglycans (the main organic component of medullary bone) (around 500 °C), the onset of crystal coarsening is delayed in cortical bone (until 600 °C, compared to 400 °C in medullary bone). This delay is likely due to the fact that apatite crystals in cortical bone are integrated into collagen fibrils, and the organic byproducts, generated after thermal decomposition of collagen, may be strongly adhered to the surface of the crystals, thereby inhibiting their growth.^16^ On the other hand, ζ-potential measurements show that the surface of cortical and medullary bone is negatively charged, and upon thermal treatment, the surface became less negative due to the loss of organic matter (see Supporting Information; Figure S5).

There are other relevant changes in bone mineral that occur during heating at higher temperatures (>600 °C). There is a notable reduction of carbonate groups in apatite and an increase in hydroxyl groups (observed by changes in the intensity of associated IR bands), as the bone mineral converts from carbonate apatite to hydroxyapatite at high temperatures (>500 °C).^15,16,25^ Moreover, under these conditions, during crystal coarsening foreign ions such as Na^+^ and Mg^2+^ diffuse out from the apatite crystal lattice and accumulate on the crystal surfaces (i.e., forming MgO nanoparticles) modifying their surface chemistry.^16,26^

Overall, these results demonstrate that thermal treatments produce extensive changes in the surface area and porosity (Figure 2, Table S2), surface chemistry, and charge of the bone mineral (Figure S5). These transformations are primarily due to the loss of organic components by the thermal decomposition and, second, to the sintering of the mineral. These changes are notably different for the two types of bone studied as they mainly depend on the organic matrix composition and the interrelation of the organics with the mineral.^16^ Moreover, these changes greatly affect the mineral reactivity, as discussed below.

**Figure 2:**
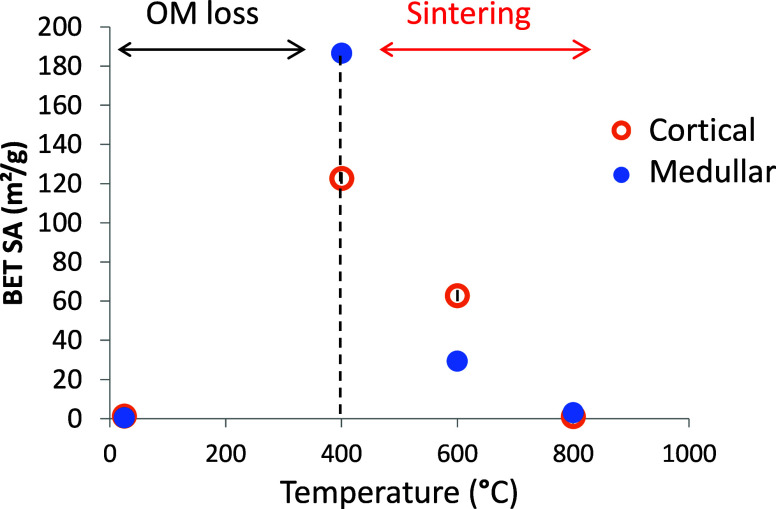
BET N_2_ surface area of cortical and medullary bone samples treated at different temperatures. The initial increase of surface area is due to microporosity generation after organic matter (OM) loss by combustion. At higher temperatures (>600 °C), there is a reduction in surface area due to the mineral sintering (crystal coarsening).

#### 3.1.2 Bone Porosity and Surface Area

Changes in bone microporosity and surface area in cortical and medullary bone upon heating were studied by N_2_ adsorption isotherms. BET isotherms data (Table S2 and Figure 2) show that the surface area of untreated bone (degassed at 100 °C) is rather low (1.47 m^2^/g in CB and 0.55 m^2^/g in MB) and increases with treatment temperature, reaching a maximum value at 400 °C (122.8 m^2^/g in CB and 186 m^2^/g in MB). Subsequently, it decreases slightly after heating the bone at 600 °C (62.81 m^2^/g in CB and 29.47 m^2^/g in MB) and becomes very low again in bone samples treated at 800 °C (1.04 m^2^/g in CB or 3.09 m^2^/g in MB). The initial increase in surface area can be attributed to the loss of organic matter by combustion and subsequent generation of microporosity. On the other hand, the decrease of surface area at higher temperatures (>600 °C) is attributed to coarsening of apatite crystal during mineral recrystallization and sintering.^16^ The greater values of BET surface area in medullary bone, compared to cortical bone at 400 °C (186 m^2^/g and 123 m^2^/g, respectively), can be attributed to its more porous structure and smaller crystal size. In any case, these results are consistent and comparable with other studies using fish, cow or chicken bone, which also reported similar surface area (80–130 m^2^/g) for bone treated at intermediate temperatures.^27–29^

### 3.2 Bone Reactivity

#### 3.2.1 Bone Mineral Dissolution

Bone mineral dissolution was studied by exposing cortical and medullary bone samples (treated at different temperatures) to diluted hydrochloric acid solution (1% HCl). The concentrations of Ca ([Ca]) and other cations released to the solution were measured using ICP-OES (Figure 3). The mineral dissolution rate was always higher for medullary bone ([Ca] 50 mg/g; MB25 °C) compared to that of cortical bone ([Ca] 17 mg/g; CB25 °C) (Figure 3A). There were also well-defined changes in bone mineral solubility in thermally treated samples. In cortical bone, [Ca] in solution remains nearly constant for samples treated up to 200 °C (17 mg/g). However, [Ca] in solution increases for samples treated at higher temperatures, reaching a maximum value for those treated at 500 °C (33 mg/g) to decrease again to a basal value (19 mg/g) for samples treated at higher temperatures (>600 °C). In medullary bone, [Ca] in solution remains nearly constant for samples treated up to 200 °C, followed by a continuous increase with treatment temperature, peaking at 600 °C (143 mg/g), before decreasing again at higher temperatures (>600 °C). Overall, the dissolution rate of medullary bone mineral was significantly higher than that of cortical bone (>5-fold), particularly for samples treated at mid-range temperatures (500–700 °C).

**Figure 3:**
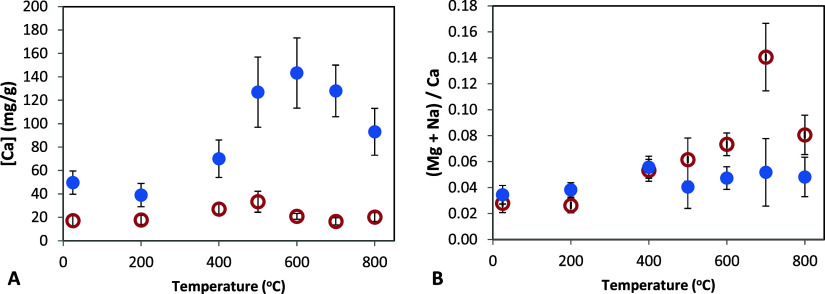
Bone mineral dissolution induced by diluted acid solution (1% HCl) for bones treated at temperatures ranging from 25 °C (untreated) to 800 °C, followed by ICP-OES. (A) [Ca] concentration in solution (after 10 min) for cortical (red open circles) and medullary (blue solid circles) bone samples treated at different temperatures. (B) ([Mg] + [Na])/[Ca] ratio in solution (after 5 min) for cortical and medullary bone samples treated at different temperatures.

For untreated bones, the higher solubility of medullary bone mineral, compared to that of cortical bone mineral, can be attributed, in part, to the smaller crystal size of apatite.^3,8^ However, after heating, the crystal sizes in both tissues became comparable and/or even greater in the medullary bone. Thus, the differences in crystal size cannot explain the observed differences in the solubility of the mineral. Cortical bone mineral is formed by apatite crystals integrated within the collagen fibrils and coated by acidic proteins (osteocalcin) strongly bound to their surface, protecting the mineral from dissolution.^9,22^ In contrast, in medullary bone, apatite crystals are embedded in an organic matrix rich in proteoglycans, which appears to be more permeable to the acidic solution.^19^ Therefore, the different organic matrix compositions and associations with the mineral in both tissues could explain the large differences in bone mineral solubility. The high solubility of medullary bone plays a crucial biological role as this bone acts as a calcium reservoir in laying hens and is the primary source of calcium for the rapid mineralization of the eggshell.^4^

For thermally treated bones, the observed changes in mineral dissolution with the treatment temperature mirror changes of other bone properties. On one hand, the significant increase in bone solubility of samples treated at mid-range temperatures (400–600 °C) is related to the loss of organic components, which protected the mineral against dissolution.^30^ On the other hand, the decrease of mineral solubility at higher temperatures (>600 °C) is due to the rapid increase of the crystal size (coarsening) during mineral sintering, reducing the surface area and making the mineral less reactive. Interestingly, the changes in mineral solubility with the treatment temperature are less pronounced in samples derived from cortical bone than medullary bone. The lower solubility, and smaller changes with temperature in cortical bone mineral solubility, suggests that the organic material in cortical bone (mostly collagen and NCP proteins), even after thermal degradation, remains more strongly attached to the crystal surfaces and is more effective at protecting the mineral from dissolution than that of medullary bone. These findings are consistent with the results of Dominguez Gasca et al.^9^ Therefore, bone mineral dissolution is controlled by surface area, crystal size, and, more importantly, the organic matrix/mineral interaction.

Other interesting changes occur during bone dissolution, as observed through various techniques. XRD data show a gradual increase in apatite crystallite size over time once the crystals are exposed to the diluted acid solution (Figure S6). This effect is more evident in bone samples treated at higher temperatures (>600 °C). For example, in cortical bone treated at 700 °C, the crystallite size increases from 63 to 130 nm (after 20 min in the diluted acid solution) (Figure S6B). This may seem counterintuitive, as one might expect dissolving crystals to become smaller. However, it is important to note that XRD measures an average crystal size. Thus, the observed increase in apatite crystallite size during mineral dissolution indicates that smaller crystals are preferentially dissolved (due to their higher surface to volume ratio, making them more reactive and soluble), whereas larger crystals remain intact,^9,30^ explaining the observed increase in the (average) crystallite size.

On the other hand, Figure 3B shows that during mineral dissolution, for untreated cortical or medullary bone, the (Mg + Na)/Ca ratio in solution is approximately ∼0.03, indicating a ∼3% ionic substitution of Ca for these other ions in the apatite structure. However, for bone samples treated at higher temperatures (>500 °C), the (Mg + Na)/Ca ratio is notably higher (up to 0.14) and, over time, it decreases to a basal value of 0.03. As described previously, during apatite sintering (and crystal coarsening) in bone samples treated at high temperatures (>500 °C), Na and Mg ions diffuse out from the apatite crystal lattice and accumulate on the crystal surfaces.^16^ Thus, initially, the outer layer of crystals (enriched with Na and Mg ions) is preferentially dissolved and later, the dissolution process reaches the ion composition of the apatitic core (with (Mg + Na)/Ca ratio of 0.03). This is evident in cortical bone treated at higher temperatures (>600 °C), as shown in Figures 3B and S6A. This selective removal of ions also affects the apatite unit cell parameters in cortical bone. In fact, during mineral dissolution, the c-parameter of the apatite unit cell (determined by XRD) increases with time (from 6.8828 to 6.8866 Å; Figure S6C). This is likely caused by the selective removal of Mg ions (with smaller ionic radius), which are replaced by Ca ions (with larger ionic radius) in the apatite structure, or to the selective removal of Mg rich outer regions, leading to an increase of the apatite unit cell dimensions in either case. Because Ca and Na cations have a very similar ionic radius, the exchange of Na ions for Ca ions should not significantly alter the unit cell parameters. The large number of ionic substitutions (i.e., Mg, Na, CO_3_) in apatite nanocrystals makes bone less stable and much more soluble than highly crystalline geological apatite,^8,31,32^ facilitating the selective removal of this outer region.

#### 3.2.2 Effect of Bone Thermal Treatment on the Bone Adsorption Potential

We studied how changes in bone mineral characteristics, induced by heating at different temperatures, influence heavy metal adsorption (using the Pb cation as a model adsorbate). Pb stabilization/immobilization/adsorption onto bone samples immersed in a Pb­(NO_3_)_2_ solution was indirectly monitored by measuring the Pb concentration remaining in the solution after exposure and was directly determined by analyzing exposed bone samples.

Pb removal by the bone samples was a very rapid and efficient process (Figure S7). In the presence of bone, the Pb concentration in solution rapidly decreased, reaching a plateau within the first hour and remaining constant thereafter. An exception was observed in some of the samples of cortical bones immersed in solutions containing 150 and 250 ppm of Pb (Figure S7A–C), which needed up to 3 h to stabilize.

Pb adsorption on bone at equilibrium, measured as *Q*_e_, varied depending on the type of bone used (cortical versus medullary), treatment temperature, and initial Pb concentration in the solution (Figures 4 and S8A–D). Figure 4 displays Q_e_ values for bone samples immersed in 150 ppm Pb solution (for other Pb concentrations tested (25- 250 ppm) see Figure S8). In all cases: (1) the amount of adsorbed Pb on bone increased with the initial Pb concentration in the solution in contact with bone samples, (2) Pb adsorption was higher in thermally treated bones compared to untreated ones, and (3) for a given concentration of Pb, the adsorption of this cation was significantly higher in medullary bone than in cortical bone, except in the case of untreated bones, where the differences in Pb adsorption between the two types of bones were not statistically significant.

**Figure 4:**
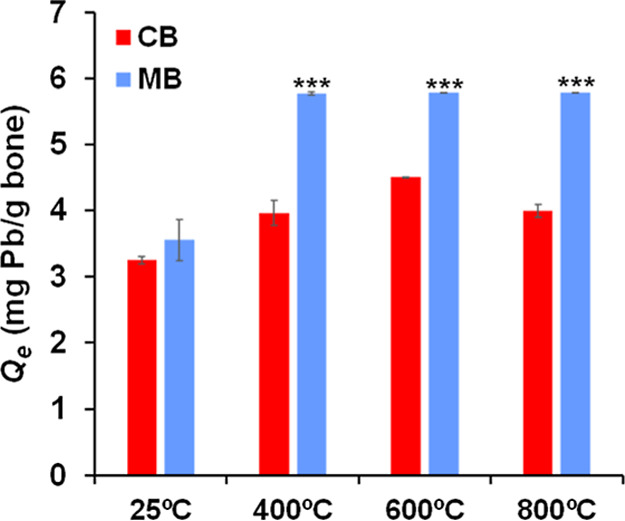
Pb adsorbed on cortical (CB) and medullary bone (MB) samples, treated at different temperatures, at equilibrium, per unit mass. This graph shows Pb values for bone samples immersed in Pb­(NO_3_)_2_ solutions containing 150 ppm Pb. Statistical differences in *Q*_e_ between cortical bones and medullary bones untreated and treated at 400 °C, 600 °C, and 800 °C were highly significant, (***) indicates *p* < 0.001. Data for samples immersed in Pb­(NO_3_)_2_ solutions containing other Pb concentrations are provided in Figure S7.

The amount of adsorbed Pb per amount of bone sample (*Q*_e_), derived from the adsorption isotherms (Figure S9), shows a nonlinear relationship with the amount of nonadsorbed Pb (*C*_e_), displaying an S-shaped curve. The *Q*_e_ values increase slowly initially, then rise exponentially and stabilize thereafter. The experimental data were adjusted to the Langmuir (L), Freundlich (F), and Langmuir–Freundlich (LF) models. The Langmuir model considers that adsorption occurs in homogeneous monolayers, where the adsorbate is equally attracted to all sites; the Freundlich model considers that adsorption occurs on heterogeneous surfaces in multiple layers; and the Langmuir–Freundlich (LF) model combines the Langmuir model at low absorbate concentrations and the Freundlich model at higher concentrations, making it particularly suited for studying adsorption on heterogeneous and porous materials such as bone.^33–35^ Only the LF model fits our experimental data (Figure S9 and Table S3). Parameters for the Langmuir–Freundlich model are extracted from Table S3 and displayed in Table 1.

**Table 1:** Absorption Isotherm Analyses. Parameters Obtained by Fitting the *Q*_e_ Data versus *C*_e_ in Figure S6 to a Langmuir–Freundlich Model, where *Q*_e_ (mg Pb/g Bone) Is the Amount of Adsorbed Pb per Mass Unit of Adsorbent at a Given Pb Concentration in Solution, *Q*_max_ (mg Pb/g Bone) Is the Maximum Pb Loading Capacity of the Bone Sample, *K*_LF_ (L/mg) Is the Affinity Constant, and *r* Is the Cooperativity Coefficient

	*K*_LF_ (L/mg)	*Q*_max_ (mg/g)	*r*	*R* ^2^
CB25 °C	0.01	17.5 ± 0.7	1.4 ± 0.2	0.994
CB400 °C	0.009 ± 0.002	26.6 ± 3.4	1.32 ± 0.4	0.973
CB600 °C	0.07 ± 0.02	116.9 ± 8.1	0.8 ± 0.2	0.976
CB800 °C	0.015 ± 0.002	53.2 ± 1.8	1.35 ± 0.2	0.981
MB25 °C	0.008	16.2 ± 0.8	2.5 ± 0.7	0.973
MB400 °C	0.347 ± 0.08	34.3 ± 1.2	0.64 ± 0.08	0.995
MB600 °C	1.0 ± 0.3	132.0 ± 5.4	0.52 ± 0.08	0.989
MB800 °C	0.013 ± 0.002	100.1 ± 5.1	0.9 ± 0.1	0.992

The maximum Pb adsorption capacity values (*Q*_max_), determined from the LF model, confirms that (1) heating both cortical and medullary bones up to 600 °C increases their adsorptive ability, and (2) thermally treated medullary bone exhibits greater adsorption capacity than cortical bone. *Q*_max_ data values for Pb adsorption of thermally treated cortical and medullary chicken bones, shown in Table 1, are considerably higher than those previously reported for other materials, such as phosphate rocks (20 mg/g,^36^), and are comparable to those of poorly crystalline apatite,^7,37^ activated carbon (120–160 mg/g,^38^), or chicken bone char (116–132 mg/g;^29^ this study 117–135 mg/g). Also, chicken bone could be a more energy- and cost-efficient alternative (at least for Pb absorption) than traditional activated carbon, that requires significantly higher temperatures (>900 °C) to be produced.

Interestingly, when the Pb concentration in the solution increased up to 4500 ppm, the adsorption capacity of Pb on CB600 °C and MB600 °C becomes comparable (Figure S10). Thus, although medullary bone has a higher surface area and adsorbs a greater amount of Pb at lower concentrations, it becomes saturated at high Pb levels. Cortical bone, however, retains available active sites, which could be contributing to the continued Pb binding, thus delaying bone saturation. This hypothesis is consistent with observations from other authors, which indicate that free active sites play a crucial role in the adsorption process.^33,39^ When Pb adsorption data are plotted as adsorption percentage for each initial Pb concentration in solution (Figure S11 and Table S4), the saturation of the different types of bone samples, considered when the bone can no longer adsorb 100% of the Pb in solution, becomes evident. The data confirm that untreated cortical and medullary bones are unable to adsorb 100% of Pb in solution, even at the lowest Pb concentrations, indicating that these materials saturate easily. However, the corresponding thermally treated bones (e.g., at 600 °C) achieve 100% Pb adsorption up to relatively high Pb concentrations in solution (3000 ppm), indicating that they reach saturation at much higher Pb concentrations and are therefore significantly more efficient adsorbents.

### 3.3 Pb Adsorption Mechanism: Insights from the Mineralogical Characterization of the Precipitates

The analyses by XRD, SEM, TEM, and EDX of untreated and thermally treated bone samples exposed to solutions containing low concentrations of Pb (<400 ppm) and high Pb concentrations (1450, 4500 ppm) provide additional information to better understand the Pb adsorption mechanism.

XRD analysis of bone samples exposed to low Pb concentrations shows that apatite was the only mineral phase detected. In contrast, in bone samples exposed to higher Pb concentrations, additional mineral phases, identified as cerussite (PbCO_3_), hydrocerussite (Pb_3_(CO_3_)_2_(OH)_2_), and/or pyromorphite (Pb-apatite) (up to 2.5%, determined by Rietveld refinement), were detected by XRD (Figure S12).

SEM observation of bone samples exposed to low Pb concentrations did not either show any mineral phase besides apatite, but TEM–EDX analysis evidenced that Pb was homogeneously distributed on the apatite crystal surface (Figure 5). On the contrary, SEM analyses of bone samples (25 °C) exposed to higher Pb concentration show the formation of new solids in the form of spheruliths, about 1–2 μm in diameter, scattered on the apatite crystals surface. Samples treated at higher temperatures (400 °C) showed the same mineral deposits covering the apatite surface (spheruliths of 2–5 μm in diameter) but with higher density (Figure 5A). Spheruliths were made of smaller flaky nanocrystals and that were identified as nanocrystalline hydrocerussite by XRD analyses (with a calculated crystallite size of 13–22 nm). Identically, XRD was used in all samples referred below to identify the new Pb-bearing solids. Bone samples treated at higher temperatures (600 °C) and higher Pb concentration (4500 ppm Pb) showed radial aggregates made of long prismatic cerussite crystals (>20 μm long; Figure 5B). There were also granular pyromorphite nanocrystals coating the apatite surface and, in some instances, pyromorphite appears as massive aggregates associated with platy-like hydrocerussite crystals (Figure 5C). Pyromophite produced broad XRD peaks due to its nanocrystalline nature (the calculated crystallite size was 14 nm). Cortical bone treated at 800 °C (exposed at 4500 ppm Pb) is completely covered with very thin hexagonal plate-like hydrocerussite crystals (about 2–5 μm wide and 150 nm thick). The previous data and information is used below to discuss potential Pb adsorption mechanisms.

**Figure 5:**
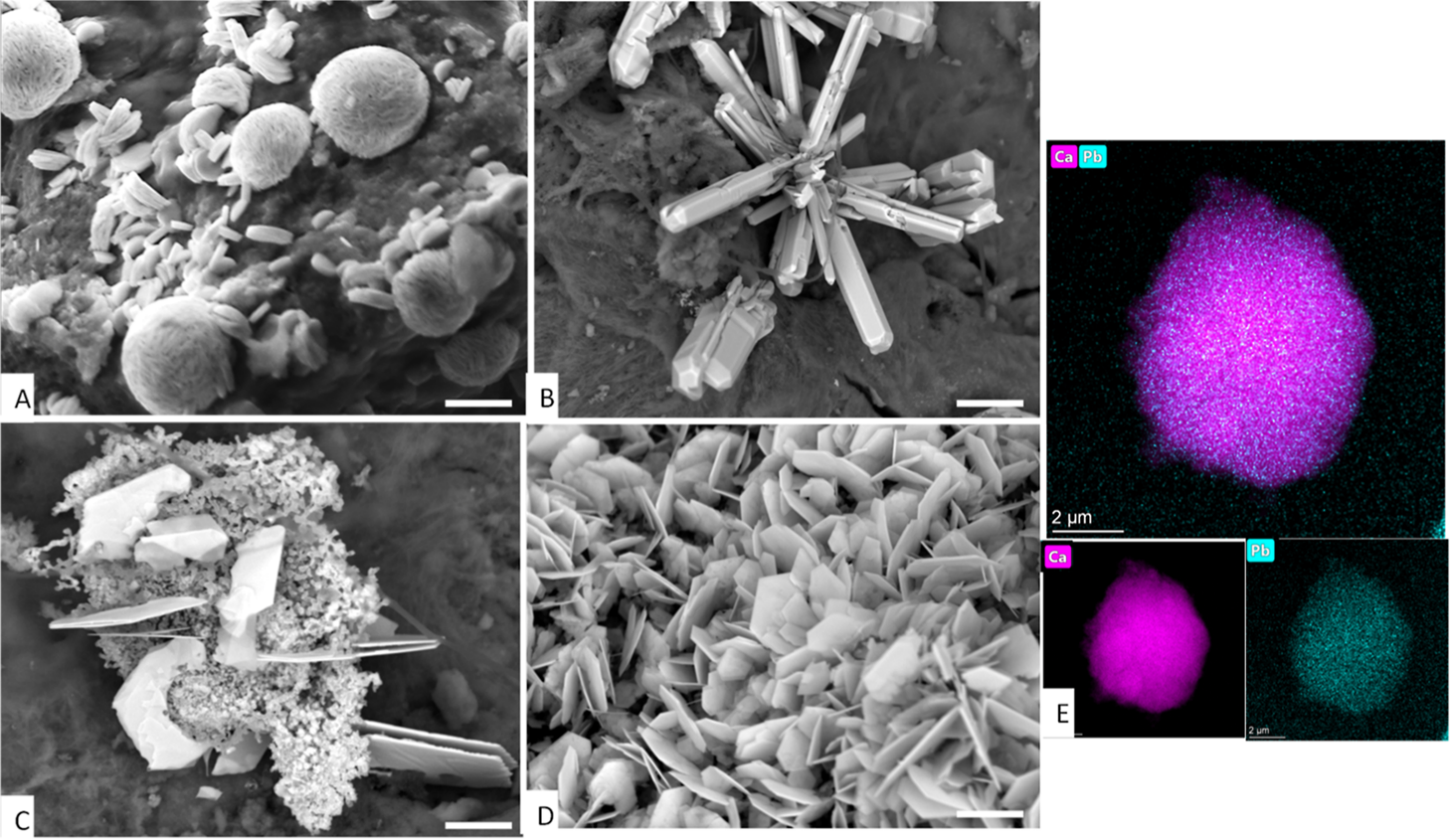
BSE-SEM images of mineral precipitates forming on bone samples treated at different temperatures and exposed to a Pb-bearing solution: (A) the surface of cortical bone (treated at 400 °C and exposed to 1450 ppm Pb solution) is decorated with spherulites made of flaky hydrocerussite nanocrystals; (B) the surface of cortical bone (600 °C and exposed to 4500 ppm Pb solution) show radial aggregates made of long prismatic cerussite crystals and hexagonal plate-like hydrocerussite crystals within a mass of pyromorphite nanocrystals (C); (D) the surface of cortical bone treated at 800 °C (and exposed to 4500 ppm Pb solution) is fully covered by thin hexagonal plate-like hydrocerussite crystals. (E) TEM–EDX elemental mapping of an apatite crystal from medullary bone treated at 600 °C (and exposed to a 150 ppm Pb solution). Scale bars: A, E: 2 μm; B: 10 μm; C, D: 5 μm.

Pb can be adsorbed onto bone through several, nonexclusive mechanisms: (a) Pb ions complexation with the organics and/or electrostatic interaction with mineral surfaces, (b) ionic exchange of Pb into the apatite structure, and (c) mineral precipitation on bone surfaces by forming Pb-mineral phases. According to ζ-potential data in Figure S5, bone particle surfaces are negatively charged within the pH range (4–5) of the adsorption experiments, particularly in untreated samples or those treated at lower temperatures (i.e., due to carboxylic or hydroxyl groups in the organic matter). Therefore, electrostatic interactions or complexation can occur between the Pb cations and the: (a) organic matter (on bone samples treated at lower temperatures). In fact, Pb has been found associated with bone collagen and NCPs;^40–42^ (b) carbonate groups in the apatite mineral (also at lower temperature treatments); and (c) hydroxyl groups,^43^ which emerge at the expense of carbonate groups during recrystallization of the mineral part (from apatite to hydroxyapatite), which occurs during thermal treatment at higher temperatures.

Other relevant Pb adsorption mechanisms on bone mineral could involve the ionic exchange of Pb^2+^ for Ca^2+^ or Mg^2+^ ions in the apatite structure.^41,44,45^ Due to the differences in the size of the cations involved [Pb^2+^ (1.20 Å), Ca^2+^ (0.99 Å), and Mg^2+^ (0.65 Å)], if this ionic exchange did indeed occur, an increase in the apatite unit cell parameters would be expected.^46,47^ However, there was no consistent change in these parameters in bone samples exposed to solutions with different Pb concentrations (Table S5). No conclusive evidence can be extracted from our data about Pb^2+^ uptake into the apatite structure. In any case, whether through electrostatic interaction or ionic exchange, Pb was homogeneously distributed at the surface of the apatite particles, as confirmed by SEM–EDX or TEM–EDX elemental maps (Figure 5E).

However, the greater Pb adsorption capacity of thermally treated bone cannot be solely explained by the increased surface area produced after heating. In fact, bone samples treated at 600 and 800 °C show similar adsorption capacities (Figure 4), though the later has a much lower BET surface area (Table 1). Therefore, additional Pb adsorption mechanisms should be considered.

As reported above (Figures S12 and S6), XRD/SEM data of bone samples exposed to high Pb concentrations show the precipitation on bone surfaces of Pb-carbonates and/or Pb-apatite mineral phases (e.g., cerussite, hydrocerussite, and pyromorphite), also confirmed by ATR-FTIR (Figure S13). Figure 6 shows that there is a very good correlation between the concentration of Ca in solution (from apatite mineral dissolution) and the amount of Pb adsorbed, at least for medullary bone, indicating that the Pb adsorption is mainly driven by the mineral dissolution.

4 $${Ca}_{5}{\left({PO}_{4}\right)}_{3}\left(OH\right)+H^{+}=5{Ca}^{2+}+3{PO}_{4}^{3-}+H_{2}O$$

**Figure 6:**
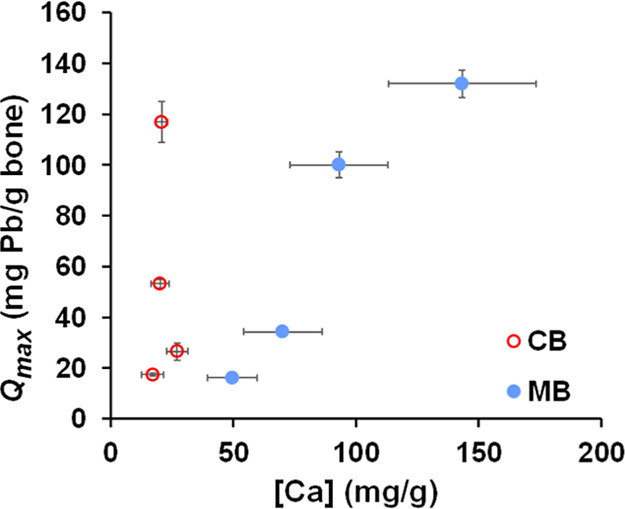
Dependence of the adsorption capacity of cortical (CB) and medullary bone (MB) samples at equilibrium per unit mass of bone (*Q*_max_) with bone mineral dissolution determined from [Ca] in solution.

These Pb-bearing mineral phases are highly insoluble (i.e., Ksp of pyromorphite is 10^–76^ or lower) and are formed through a coupled dissolution–reprecipitation reaction. The acidic pH values of the Pb­(NO_3_)_2_ solution produces the dissolution of the original bone mineral (Ca-apatite), releasing Ca and phosphate ions at the bone/solution interface, which increases supersaturation and induces the precipitation of highly insoluble Pb mineral phases at the bone surfaces. Other authors^5,6,36,48^ describe the precipitation of pyromorphite as the primary mechanism for Pb immobilization of apatite minerals under acidic conditions. However, the dissolution of apatite has a strong pH buffering capacity, increasing the pH to ∼7 which ultimately would limit the coupled dissolution–precipitation reaction (see apatite dissolution reaction (4); Table S1). On the other hand, as the pH increases (≥7), the concentration of dissolved phosphate decreases, favoring instead the precipitation of Pb-carbonates (i.e., cerussite, hydrocerussite).^5^ Therefore, in any case, the efficiency of Pb removal by apatitic materials seems to be mainly controlled by its dissolution rate and its strong pH buffering capacity, which allows the precipitation of different Pb-bearing mineral phases in the coupled dissolution reprecipitation process.

## 4 Conclusions

Thermal treatment of cortical and medullary chicken bones up to 800 °C produces well-defined changes in microstructure, porosity, surface area, and surface charge that substantially modify mineral reactivity. The loss of organic components during heating at lower temperatures (<400 °C) generates new porosity and modifies bone surface chemistry and charge. Treatment at higher temperatures (>400 °C) induces mineral sintering, leading to crystal coarsening, and modification of the organization of the mineral, pore distribution, and surface area. This process is influenced by the interaction of the mineral phase with the organics, that produces distinct changes in cortical and medullary bone. At higher temperatures (>600 °C), the diffusion of foreign ions (Na, Mg) from the apatite crystal lattice to the crystal surfaces alters mineral surface chemistry. These structural and chemical changes determine the different behavior of cortical and medullary bone samples during mineral dissolution and adsorption experiments.

Bone mineral solubility increases with the treatment temperature as the organic components, which protect bone mineral against dissolution, are lost, and increased porosity facilitates solution access. At the highest temperature treatments, crystal coarsening during mineral sintering reduces the mineral solubility. Medullary bone consistently exhibits higher dissolution rates than those of cortical bone under acidic conditions, reflecting its intrinsically higher mineral reactivity.

Thermal treatment markedly enhances the capacity of both the cortical and medullary bone to act as Pb sorbents.

While some Pb sorption may occur via ion complexation or electrostatic interaction with negatively charged functional groups on the mineral surface, the prevailing mechanism under acidic conditions and elevated Pb concentrations involves a coupled dissolution–precipitation process. Specifically, partial dissolution of the bone apatite promotes the release of phosphate, which subsequently induced the precipitation of highly insoluble Pb-bearing mineral phases (e.g., pyromorphite and hydrocerussite), at the bone surface, effectively immobilizing Pb. This process is further enhanced by at intermediate temperatures which increases mineral solubility (especially in medullary bone), rising phosphate in solution and promoting Pb-mineral precipitation. On the other hand, high temperature-induced mineral sintering decreases mineral reactivity by reducing surface area and dissolution rates. Overall, these findings demonstrate the potential to control physicochemical properties of bone-derived materials, by thermal treatment, enabling the valorization of chicken bone waste into specific applications such as the development of effective sorbents for heavy metal contamination remediation.

## Supplementary Material



## Notes

Data from SEM, TGA, BET, Pb adsorption, zeta-potential, XRD and FTIR analyses, data from pH of solutions, BET, L-F fit model, Pb adsorption, and XRD (PDF)
