# Supplementary material for: Heat-Induced Changes in Avian (Cortical and Medullary) Bone Mineral Reactivity, Solubility, and Adsorption Capacity
Source: ACS Omega. 2026 May 20;11(21):30744–54. doi: 10.1021/acsomega.5c12924 (PMC13234880; doi:10.1021/acsomega.5c12924)
Supplement: Supplementary file 1 [file ao5c12924_si_001.pdf]

Supporting information

Heat-induced changes in avian (cortical and medullary) bone mineral reactivity, solubility and adsorption capacity

Tamara Pozo-Gualda[a], Monica Jimenez-Carretero[a], Pablo Rodriguez-Jimenez[b], Miguel Burgos-Ruiz[b], Concepcion Jimenez-Lopez[a,*], Alejandro B. Rodriguez-Navarro[b,*]

[a] Dpto. Microbiologia. Facultad de Ciencias. 18071, Spain

[b] Dpto. Mineralogia y Petrologia. Facultad de Ciencias. Universidad de Granada, 18071, Spain

[*] Corresponding authors: Alejandro B. Rodriguez-Navarro (anava@ugr.es)[1]; Concepcion Jimenez-Lopez (cjl@ugr.es)[2]

[1] Phone: +34 958240059

[2] Phone: +34 958249833

Figure S1. A) BSE-SEM image of a tibia cross-section showing cortical bone (CB), and medullary (MB) bone, formed by isolated trabeculae partially filling the marrow cavity. B) Detail of cortical bone showing the osteons with Haversian channels (Hc) at the center, surrounded by osteocytes (small arrows). Scale bars: A) 500 µm; B) 50 µm.

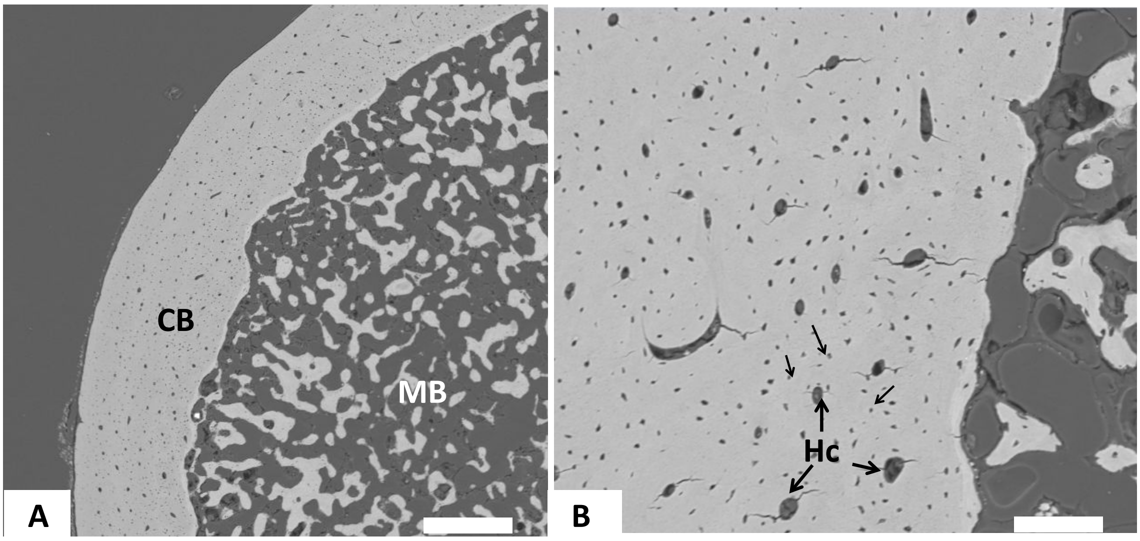

Table S1. pH values of the different $Pb(NO_3)_2$ solutions containing different Pb concentrations (initial pH) and after immersion of the different bone samples with 24 h incubation (final pH). Bone samples: Cortical bone (CB) and medullary bone (MB); untreated CB (CB25ºC), CB treated at 400ºC (CB400ºC), CB treated at 600ºC (CB600ºC), CB treated at 800ºC (CB800ºC), untreated MB (MB25ºC), MB treated at 400ºC (MB400ºC), MB treated at 600ºC (MB600ºC) and MB treated at 800ºC (MB800ºC).

Sample	Lead (ppm)	Initial pH	Final pH
CB25ºC	25	5.82 ± 0.22	7.95 ± 0.05
CB25ºC	1450	4.85 ± 0.19	5.24 ± 0.08
CB400ºC	25	5.82 ± 0.22	7.89 ± 0.05
CB400ºC	1450	4.85 ± 0.19	5.15 ± 0.07
CB600ºC	25	5.95 ± 0.5	8.5 ± 0.2
CB600ºC	1450	5.1 ± 0.04	5.3 ± 0.2
CB600ºC	4500	4.78 ± 0.04	5.00 ± 0.05
CB800ºC	25	5.95 ± 0.5	10.4 ± 0.2
CB800ºC	1450	5.1 ± 0.04	5.8 ± 0.4
CB800ºC	4500	4.78 ± 0.04	5.6 ± 0.1
MB25ºC	25	5.82 ± 0.22	7.53 ± 0.05
MB25ºC	1450	4.85 ± 0.19	5.2 ± 0.1
MB400ºC	25	5.82 ± 0.22	8.0 ± 0.3
MB400ºC	1450	4.85 ± 0.19	5.5 ± 0.1
MB600ºC	25	5.95 ± 0.5	9.8 ± 0.2
MB600ºC	1450	5.1 ± 0.04	5.6 ± 0.2
MB600ºC	4500	4.78 ± 0.04	5.3 ± 0.2
MB800ºC	25	5.95 ± 0.5	9.7 ± 0.3
MB800ºC	1450	5.1 ± 0.04	5.8 ± 0.3
MB800ºC	4500	4.78 ± 0.04	5.5 ± 0.2

Figure S2. Thermogravimetric analysis of cortical bone showing the main weight loss events due to the lost of water (25-200 ºC), organic matter (200-600 ºC) and carbonate (600-800 ºC) as well as the heat transfer (below) during these processes.

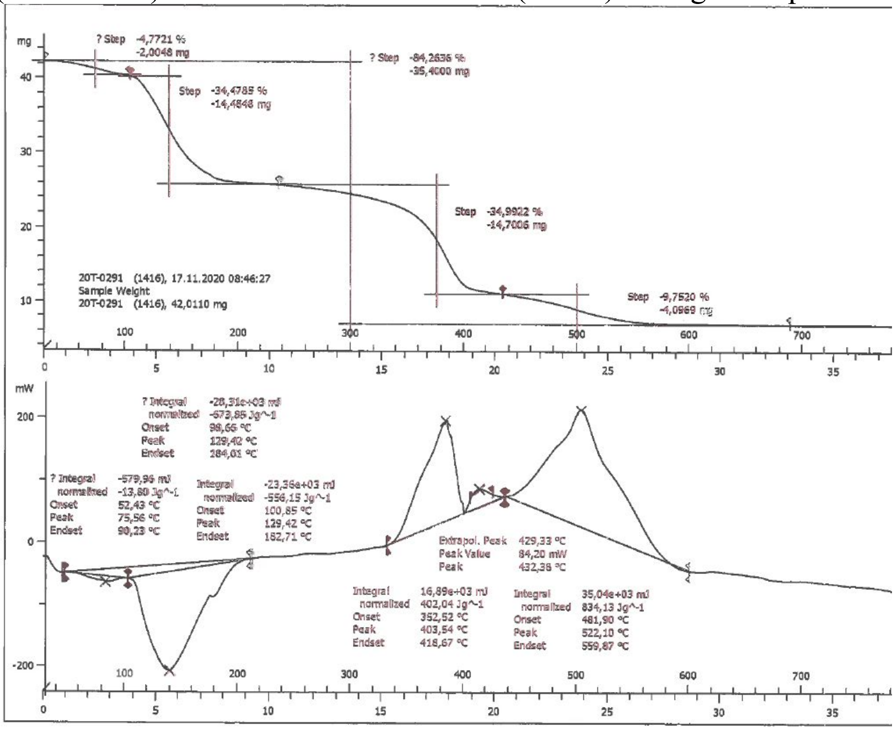

Figure S3. N_2 adsorption-desorption isotherms. (A) and (B) cortical bones (CB), untreated (CB25ºC) and thermally treated (CB400ºC, CB600ºC and CB800ºC), and (C) and (D) medullary bones (MB), untreated (MB25ºC) and thermally treated (MB400ºC, MB600ºC and MB800ºC).

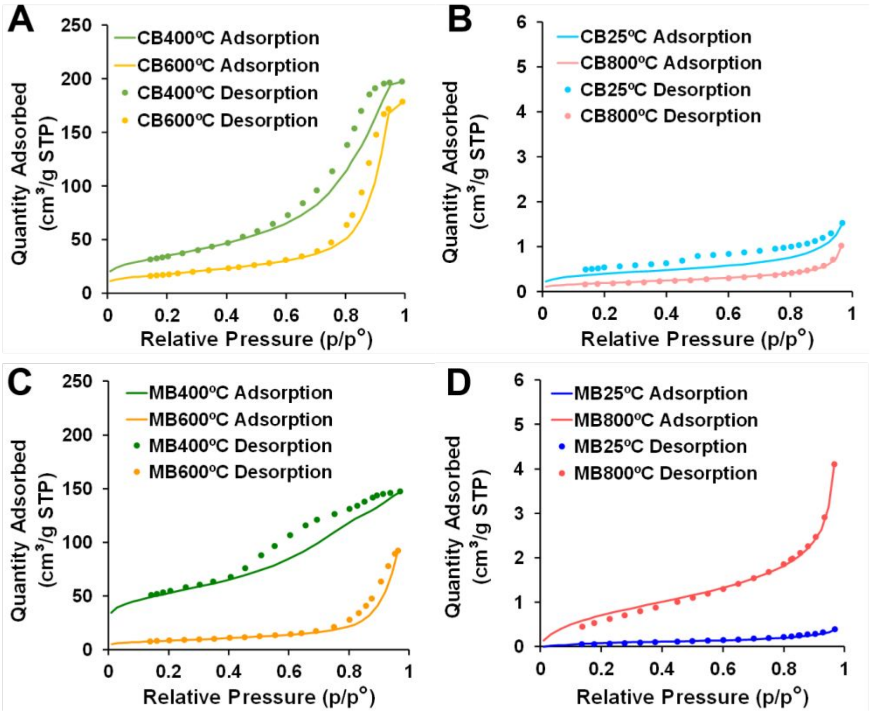

Table S2. BET surface area (BET SA), micropore area (MA), external surface area (ESA) and pore size of cortical and medullary bone samples treated at different temperatures and measured in N_2.

Bone simple	BET SA (m²/g)	t-plot MA (m²/g)	t-plot ESA (m²/g)	Pore size (nm)
CB25ºC	1.460 ± 0.004	0.16	1.30	6.4
CB400ºC	122.80 ± 0.3	2.53	120.31	9.9
CB600ºC	62.81 ± 0.04	6.49	56.33	16.9
CB800ºC	1.04 ± 0.005	-	1.34	8.9
MB25ºC	0.55 ± 0.01	-	0.77	4.4
MB400ºC	186.58 ± 0.55	40.23	146.36	5.2
MB600ºC	29.47 ± 0.02	3.23	26.25	19.3
MB800ºC	3.09 ± 0.02	-	4.60	8.2

BET data also show that micropore size increases during heating until 600 °C, and then decreases again (Table S2). All bone samples exhibit pore sizes corresponding to mesopores, ranging from 4 to 20 nm. It is noteworthy that medullary bone treated at 600 °C exhibits fewer micropores than cortical bone, possibly because sintering starts earlier (at lower temperatures) in medullary bone than in cortical bone [1]. Bone samples having very low microporosity (untreated bone or treated at 800 °C) behave like non-porous or macroporous adsorbents [type II, IUPAC classification [2]]. In contrast, samples treated at mid-range temperatures (400 - 600 °C) behave like mesoporous adsorbents [type IV, IUPAC classification [3] and demonstrate H4 type hysteresis loops, which are associated with narrow pore shapes in the form of ridges [4].

Figure S4: X-ray diffraction pattern of: (A) Cortical bones (CB25ºC, CB400ºC, CB600ºC, CB800ºC and (B) Medullary bones (MB25ºC, MB400ºC, MB600ºC andMB800ºC).

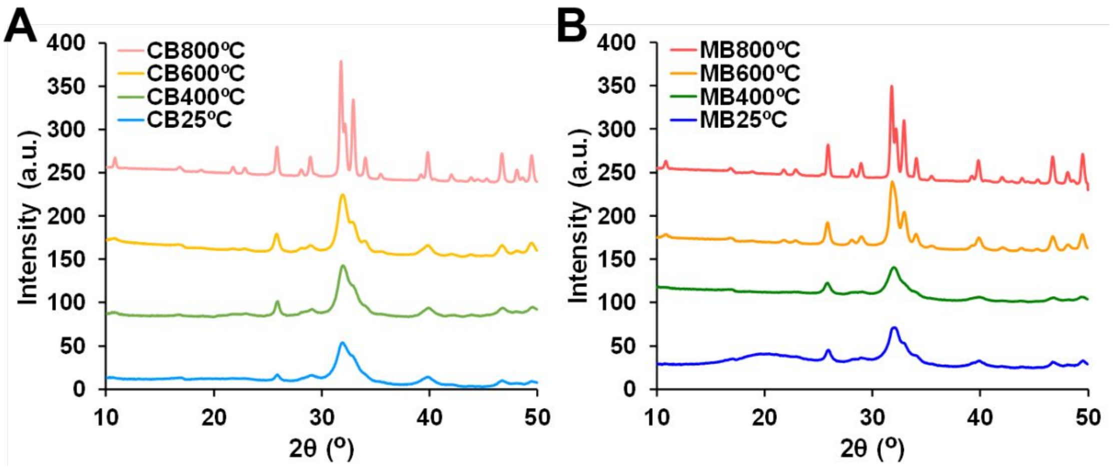

Figure S5. Zeta potential of bone samples in the pH range of 5.0 to 11.0. A) Cortical bone samples treated at different temperatures; B) Medullary bone samples treated at different temperatures.

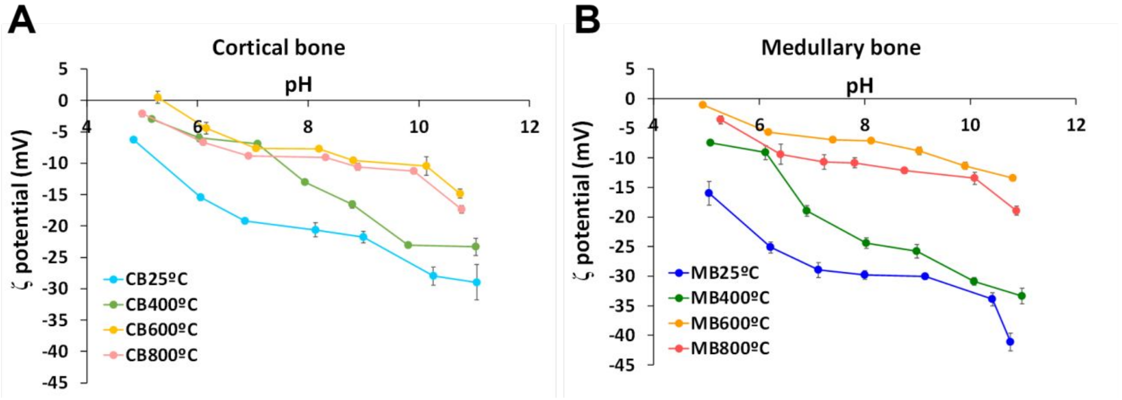

Figure S5 shows the ζ-potential for bone samples treated at different temperatures, in the pH range of 5.0 to 11.0. The ζ-potential decreases as the pH increases, due to the deprotonation of carboxylic or hydroxyl groups present in the organic matter, resulting in the negative surface charge observed within this pH range. These results are consistent with previous studies [5,6]. For cortical untreated bone, the ζ-potential decreased from ~-7 mV at pH 5 to ~-30 mV at pH 11.0. Medullary untreated bone had a slightly more negative surface charge than cortical bone (from ~-16 mV at pH 5 to ~-40 mV at pH 11.0.), which is likely due to the presence of proteoglycans [7]. With increasing temperatures, the surface charge became less negative due to the loss of organic matter as described by other authors [1,8]. Changes is bone surface charge after heating at higher temperatures could be also due to the diffusion of Na and Mg ions to the surface and the formation of CaO and MgO as well as the loss of carbonate groups.

Figure S6. Changes of bone mineral during acid induced dissolution. (A) ([Mg] + [Na]) / [Ca] ratio in solution for cortical (CB) and medullary (MB) bone treated at different temperaturas. Changes in apatite crystallite size (B) and unit cell parameters (C) during mineral dissolution for a cortical bone sample treated at 700 °C.

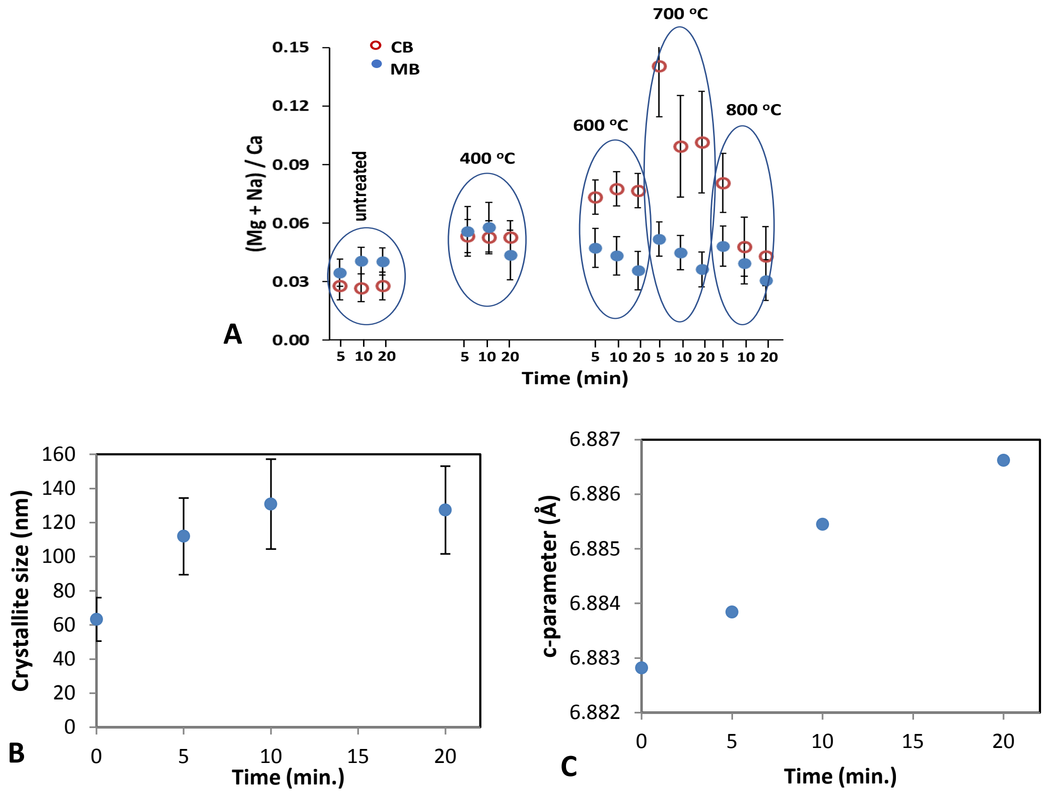

Figure S7. Kinetics of Pb adsorption. (A) to (D) cortical bones (CB) and (E) to (H) medullary bones (MB) incubated with $Pb(NO_3)_2$ solutions containing 25 ppm, 50 ppm, 75 ppm, 150 ppm and 250 ppm of Pb. (A) untreated CB (CB25ºC), (B) CB treated at 400ºC (CB400ºC), (C) CB treated at 600ºC (CB600ºC) and (D) CB treated at 800ºC (CB800ºC). (E) untreated MB (MB25ºC), (F) MB treated at 400ºC (MB400ºC), (G) MB treated at 600ºC (MB600ºC) and (H) MB treated at 800ºC. (MB800ºC). When time zero adsorption was compared to the later times, differences were statistically significan, (***) indicat es $p < 0.001$.

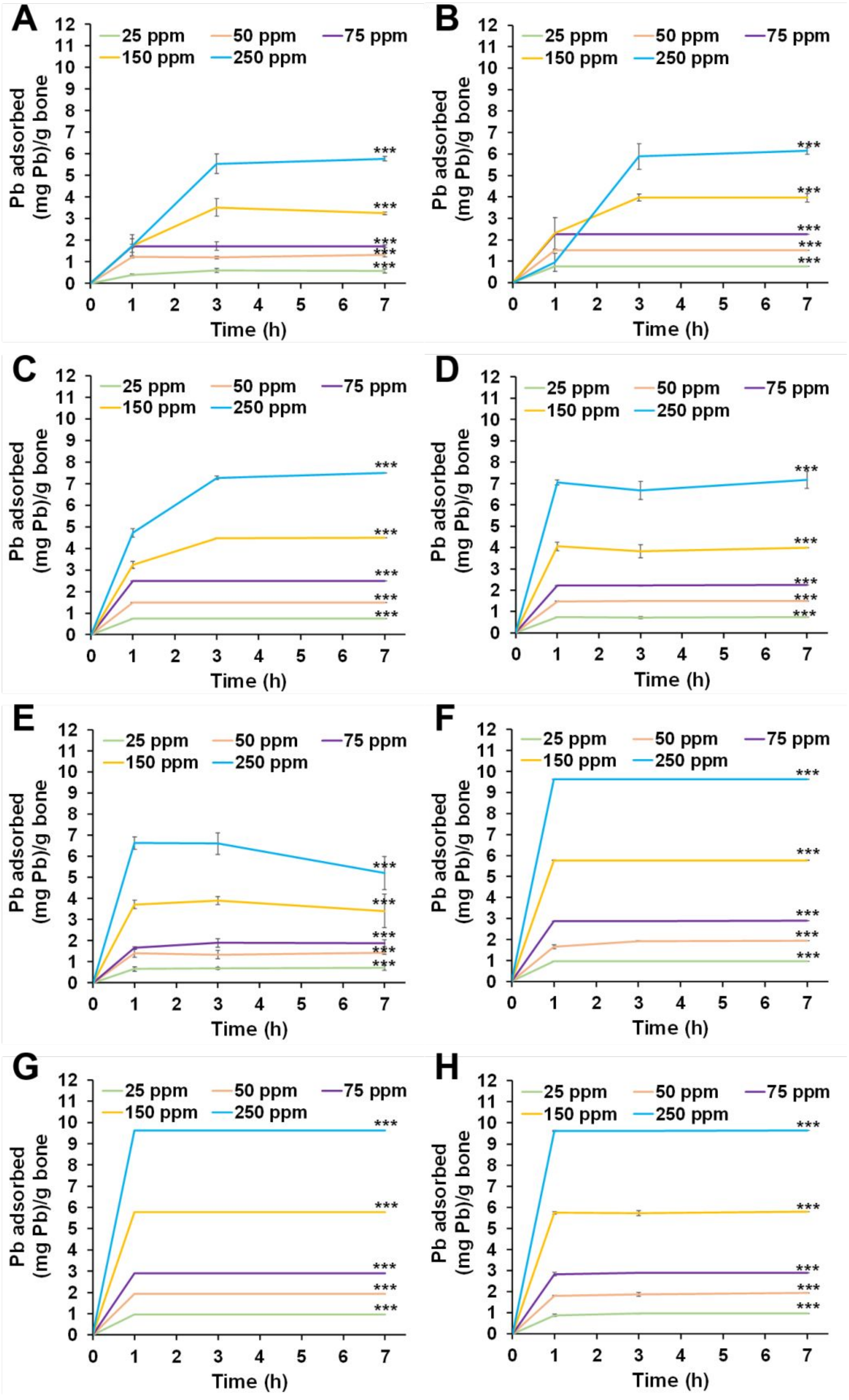

Figure S8. Pb adsorbed on cortical (CB) and medullary bone (MB) samples, treated at different temperatures, at equilibrium, per unit mass, that were immersed in $Pb(NO_3)_2$ solutions containing (A) 25 ppm Pb, (B) 50 ppm Pb, (C) 75 ppm Pb and (D) 250 ppm Pb. Statistical differences in Q_e between cortical bones and medullary bones untreated and treated at 400ºC, 600ºC and 800ºC were highly significant, (***) indicates $p < 0.001$.

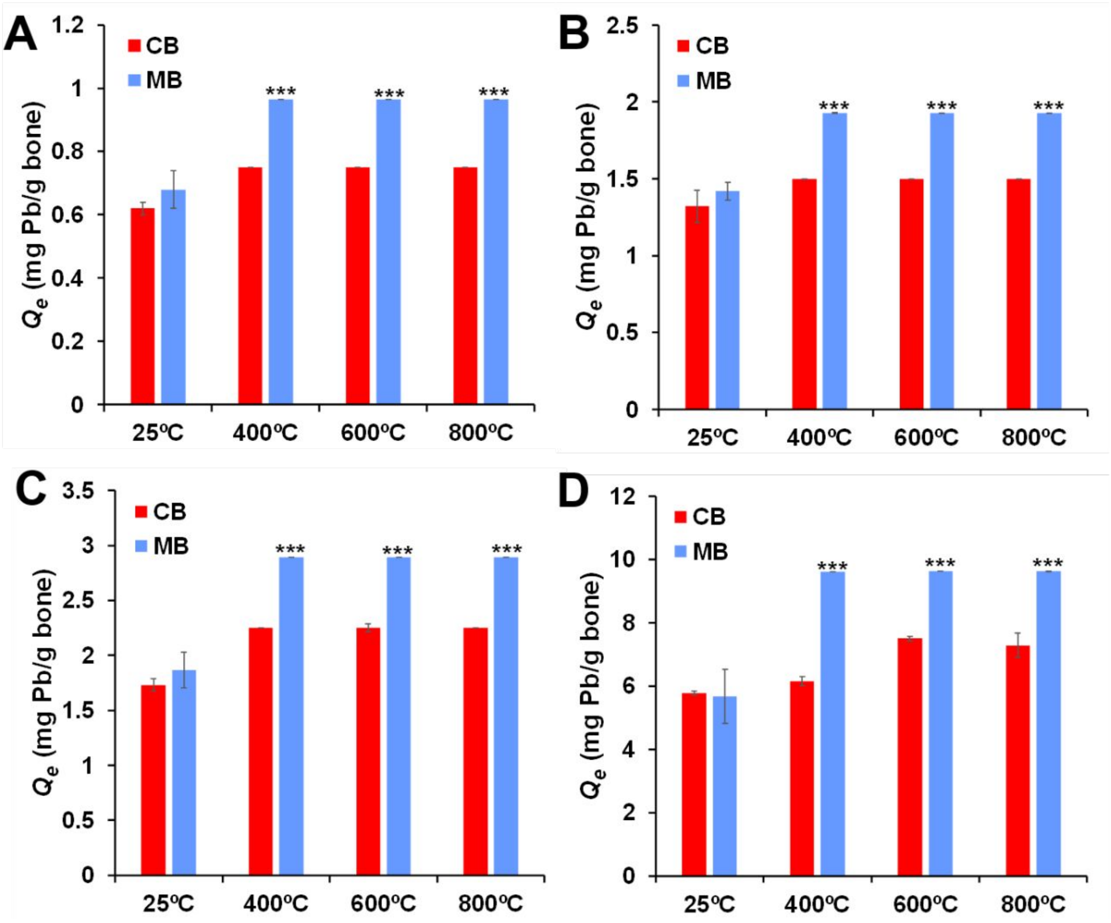

Figure S9. Experiment adsorption isotherms of Pb. (A) to (D) cortical bones (CB) and (E) to (H) medullary bones (MB) incubated with $Pb(NO_3)_2$. (A) untreated CB (CB25ºC), (B) CB treated at 400ºC (CB400ºC), (C) CB treated at 600ºC (CB600ºC) and (D) CB treated at 800ºC (CB800ºC). (E) untreated MB (MB25ºC), (F) MB treated at 400ºC (MB400ºC), (G) MB treated at 600ºC (MB600ºC) and (H) MB treated at 800ºC (MB800ºC). Exp: the experimental isotherm. F Model: the fit to the Freundlich model. LF Model: the fit to the Langmuir-Frendlich model (LF Model).

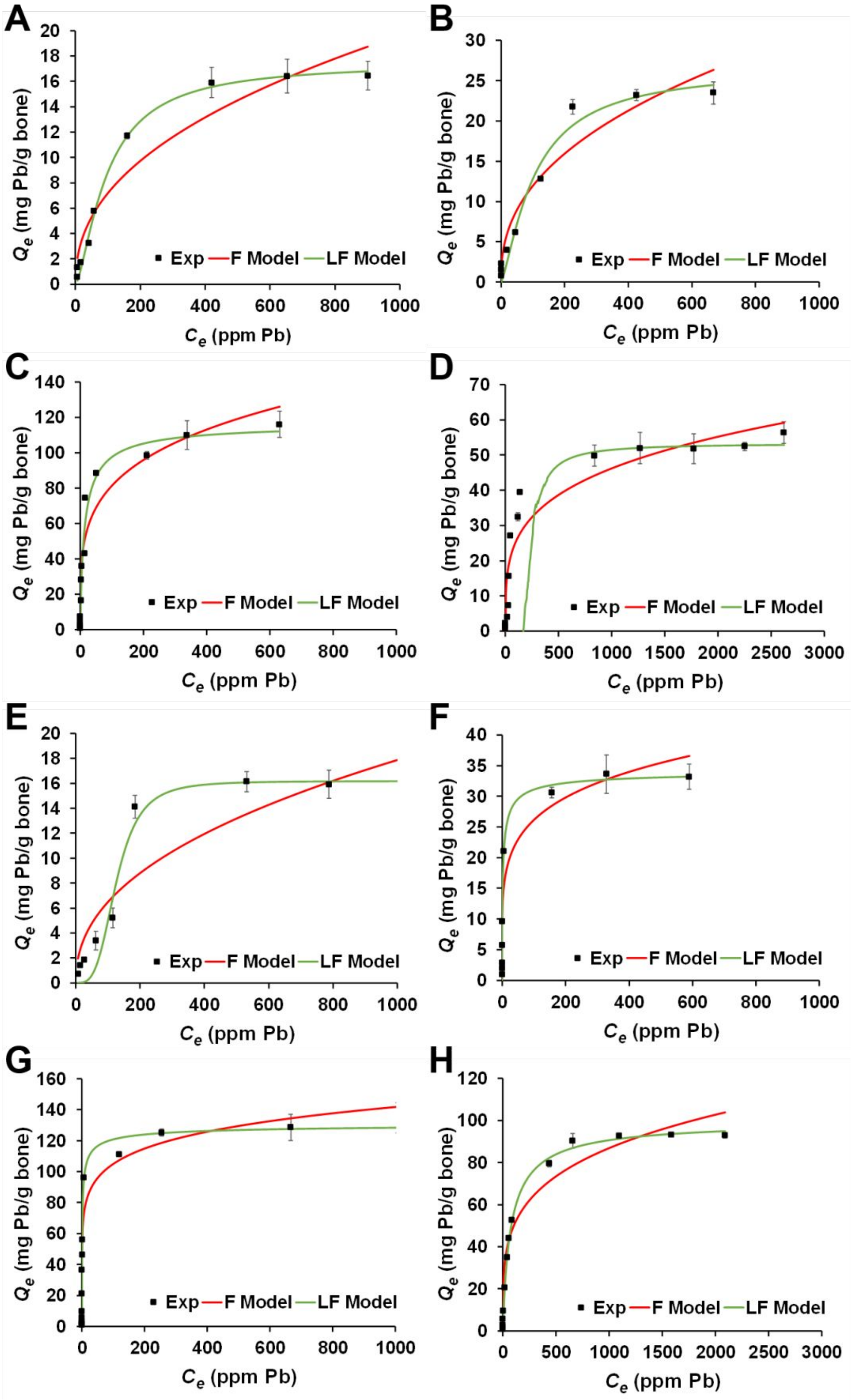

Table S3. Parameters obtained by fitting the Q_e data versus C_e in figure S5 to the Langmuir, Freundlich and Langmuir-Freundlich models. Q_e (mg Pb/g bone) is the amount of adsorbed Pb per mass unit of adsorbent at a given Pb concentration in solution and C_e (mg/L) the amount of non-adsorbed Pb at equilibrium.

	Freundlich Model			***Langmuir-Freundlich Model***				***Langmuir Model***		
	K_F (mg$^{1\text{-}1/n}$ L$^{1/n}$/g)	n	R^2	K_{LF} (L/mg)	Q_{max} (mg/g)	r	R^2	K_L (L/mg)	Q_{max} (mg/g)	R^2
**CB25ºC**	1.0 ± 0.4	2.3 ± 0.4	0.927	0.01	17.5 ± 0.7	1.4 ± 0.2	0.995	-	-	-
**CB400ºC**	1.8 ± 0.8	2.4 ± 0.5	0.932	0.009 ± 0.002	26.6 ± 3.4	1.32 ± 0.4	0.973	-9.9 ± 7.1	15.2 ± 3.7	0.48
**CB600ºC**	27.3 ± 4.2	4.2 ± 0.5	0.939	0.07 ± 0.02	116.9 ± 8.1	0.8 ± 0.2	0.976	-1.3 ± 1.2	61.9 ± 25.0	0.408
**CB800ºC**	7.9 ± 2.2	3.9 ± 0.6	0.912	0.015 ± 0.002	53.2 ± 1.8	1.35 ± 0.2	0.981	-7.6 ± 4.7	35.3 ± 5.4	0.417
**MB25ºC**	0.9 ± 0.6	2.3 ± 0.5	0.852	0.008	16.2 ± 0.9	3.5 ± 1.2	0.962	-	-	-
**MB400ºC**	10.9 ± 1.6	5.3 ± 0.8	0.949	0.347 ± 0.08	34.3 ± 1.2	0.64 ± 0.08	0.995	-13.3 ± 11.9	18.8 ± 4.9	0.241
**MB600ºC**	57.6 ± 4.7	7. 7 ± 0.9	0.962	1.0 ± 0.3	132.0 ± 5.4	0.52 ± 0.08	0.989	2.1 ± 0.4	121.6 ± 4.2	0.973
**MB800ºC**	16.3 ± 2.8	4.13± 0.4	0.968	0.013 ± 0.002	100.1 ± 5.1	0.9 ± 0.1	0.992	-1.4 ± 6.3	61.1 ± 9.0	0.500

Figure S10. Pb adsorbed on different bone samples at equilibrium per unit of mass. For the different types of bone samples (CB and MB treated at 600ºC and 800ºC) immersed in $Pb(NO_3)_2$ solutions containing: (A) 4000 ppm Pb, (B) 4500 ppm Pb. Statistical differences in Q_e between CB and MB treated at 600ºC and 800ºC were highly significant, (***) indicates $p < 0.001$.

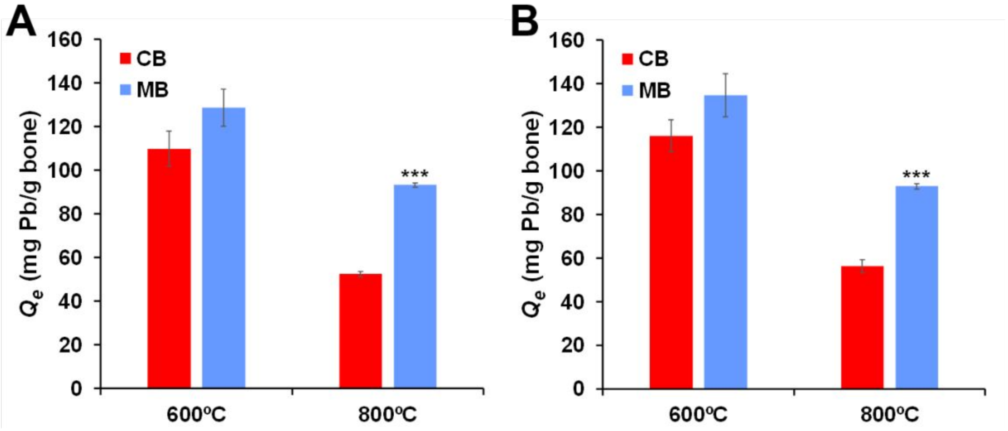

Figure S11. Percentage of Pb adsorbed on bone samples for each initial Pb concentration. (A) Cortical bones, (B) medullary bones.

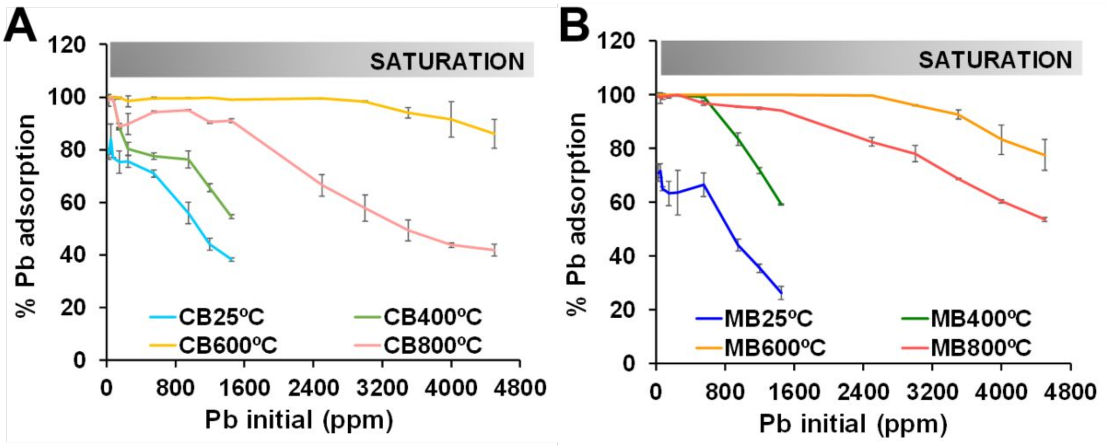

Table S4. Adsorption of Pb at equilibrium as a percentage.

[Pb] (ppm)	CB25ºC	CB400ºC	CB600ºC	CB800ºC	MB25ºC	MB400ºC	MB600ºC	MB800ºC
**25**	78.45 ± 2.16	100	99.99 ± 0.02	98.72 ± 2.22	70.96 ± 3.13	99.94 ± 0.1	100	99.59 ± 0.58
**50**	84.02 ± 5.84	99.98 ± 0.04	100	99.88 ± 0.18	71.74 ± 2.56	99.72 ± 0.34	100	98.63 ± 1.94
**75**	76.92 ± 0.02	99.94 ± 0.10	99.99 ± 0.02	99.57 ± 0.6	65.10 ± 0.8	99.54 ± 0.38	100	99.33 ± 1.16
**150**	75.19 ± 4.28	88.18 ± 0.22	99.68 ± 0.45	88.81 ± 1.24	63.34 ± 4.54	99.73 ± 0.09	100	99.37 ± 0.59
**250**	75.49 ± 2.21	80.25± 2.44	98.41 ± 2.2	89.72 ± 3.99	63.58 ± 8.32	99.79 ± 0.10	100	99.88 ± 0.11
**550**	70.93 ± 1.34	77.43 ± 1.25	99.55 ± 0.34	94.33 ± 0.34	66.51 ± 4.34	99.13 ± 0.27	100	96.88 ± 0.9
**950**	55.83 ± 4.14	76.24± 3.21	99.62 ± 0.065	95.09 ± 0.25	44.02 ± 2.19	83.54 ± 2.3	100	95.50 ± 0.25
**1200**	44.03 ± 2.17	65.53 ± 1.49	99.64 ± 0.01	90.46 ± 0.42	35.51 ± 1.58	71.81 ± 1.16	100	95.05 ± 0.39
**1450**	38.25 ± 0.61	54.5 ± 0.76	99.07 ± 0.01	91.01 ± 0.73	26.37 ± 2.41	59.17 ± 0.24	100	94.18
**2500**			99.39 ± 0.04	66.42 ± 4.07			99.78 ± 0.03	82.47 ± 1.55
**3000**			98.29 ± 0.16	57.75 ± 4.93			96.06 ± 0.21	78.10 ± 3.01
**3500**			93.96 ± 2.03	49.32 ± 3.99			92.74 ± 1.69	68.77 ± 0.24
**4000**			91.56 ± 6.8	43.68 ± 0.96			83.35 ± 5.49	60.41 ± 0.61
**4500**			85.99 ± 5.41	41.76 ± 2.26			77.59 ± 5.7	53.54 ± 0.71

Figure S12: X-ray diffraction pattern from XPowderX software analysis of: (A) CB25ºC, (B) CB400ºC, (C) CB600ºC, (D) CB800ºC, (E) MB25ºC, (F) MB400ºC, (G) MB600ºC and (H) MB800ºC immersed in solutions with the highest concentration of $Pb(NO_3)_2$ used for each type of bone in the experiments (1450ppm for untreated and treated at 400ºC bones and 4500ppm for treated at 600ºC and 800ºC bones). HC: Peaks corresponding to hydrocerussite. C: Peaks corresponding to cerussite. P: Peaks corresponding to plumbonacrite. Peaks without symbol corresponding to hydroxylapatite.

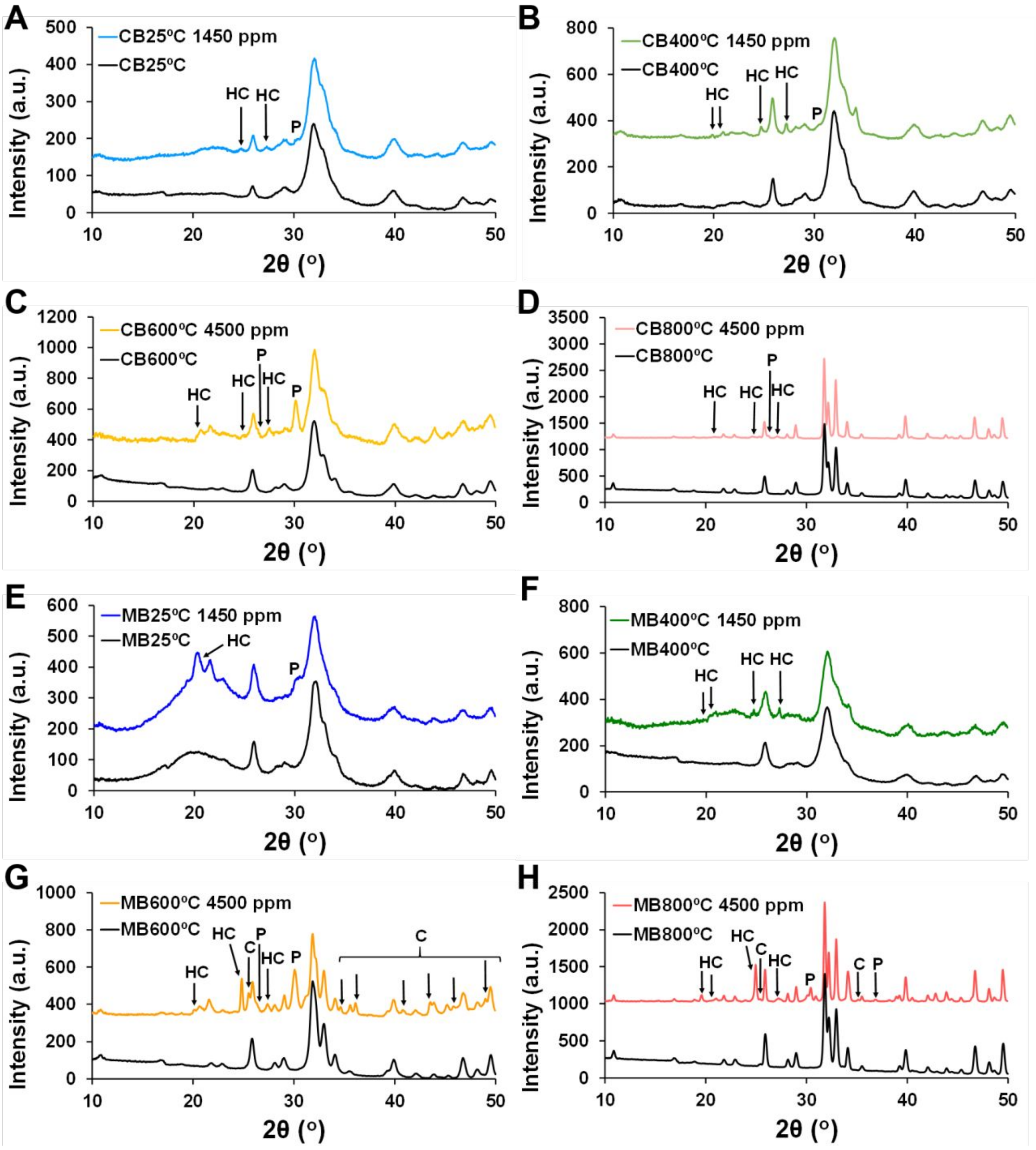

Table S5. Changes in lattice parameters (a and b) of untreated and treated bones incubated with different concentrations of Pb.

Bone	[Pb](ppm)	a (Å)	c (Å)	Bone	[Pb] (ppm)	a (Å)	c (Å)
**CB25ºC**	0	9.4528	6.9139	**MB25ºC**	0	9.3987	6.8793
	75	9.4304	6.8921		75	9.4312	6.8889
	250	9.4561	6.9121		250	9.4152	6.8887
	1450	9.4294	6.9034		1450	9.4474	6.8729
**CB400ºC**	0	9.3968	6.8913	**MB400ºC**	0	9.4083	6.8893
	75	9.4244	6.8968		75	9.4214	6.8972
	250	9.410	6.896		250	9.3985	6.8899
	1450	9.4148	6.8989		1450	9.4133	6.9033
**CB600ºC**	0	9.4169	6.8981	**MB600ºC**	0	9.4203	6.8936
	75	9.424	6.8935		75	9.4147	6.8917
	250	9.4124	6.8934		250	9.4099	6.8943
	2500	9.4153	6.8991		4500	9.4203	6.9006
	4500	9.4382	6.9184				
**CB800ºC**	0	9.4184	6.8908	**MB800ºC**	0	9.4271	6.8876
	75	9.4214	6.8897		75	9.4333	6.8871
	250	9.4214	6.8899		250	9.4175	6.8884
	4500	9.4216	6.8928		2500	9.4185	6.8885
					4500	9.4191	6.8866

Figure S13. FT-IR spectra of (A) cortical and (B) medullary bone samples untreated (CB25ºC, MB25ºC, respectively) and treated at different temperatures (CB400ºC, CB600ºC, CB800ºC, MB400ºC, MB600ºC, MB800ºC) not immersed and immersed in $Pb(NO_3)_2$ solutions containing 1450 or 4500 ppm Pb. (C) Detail showing peaks around 837 cm^{-1} and 670-680 cm^{-1}, and the main carbonate band from 1410 to 1390 cm^{-1}.

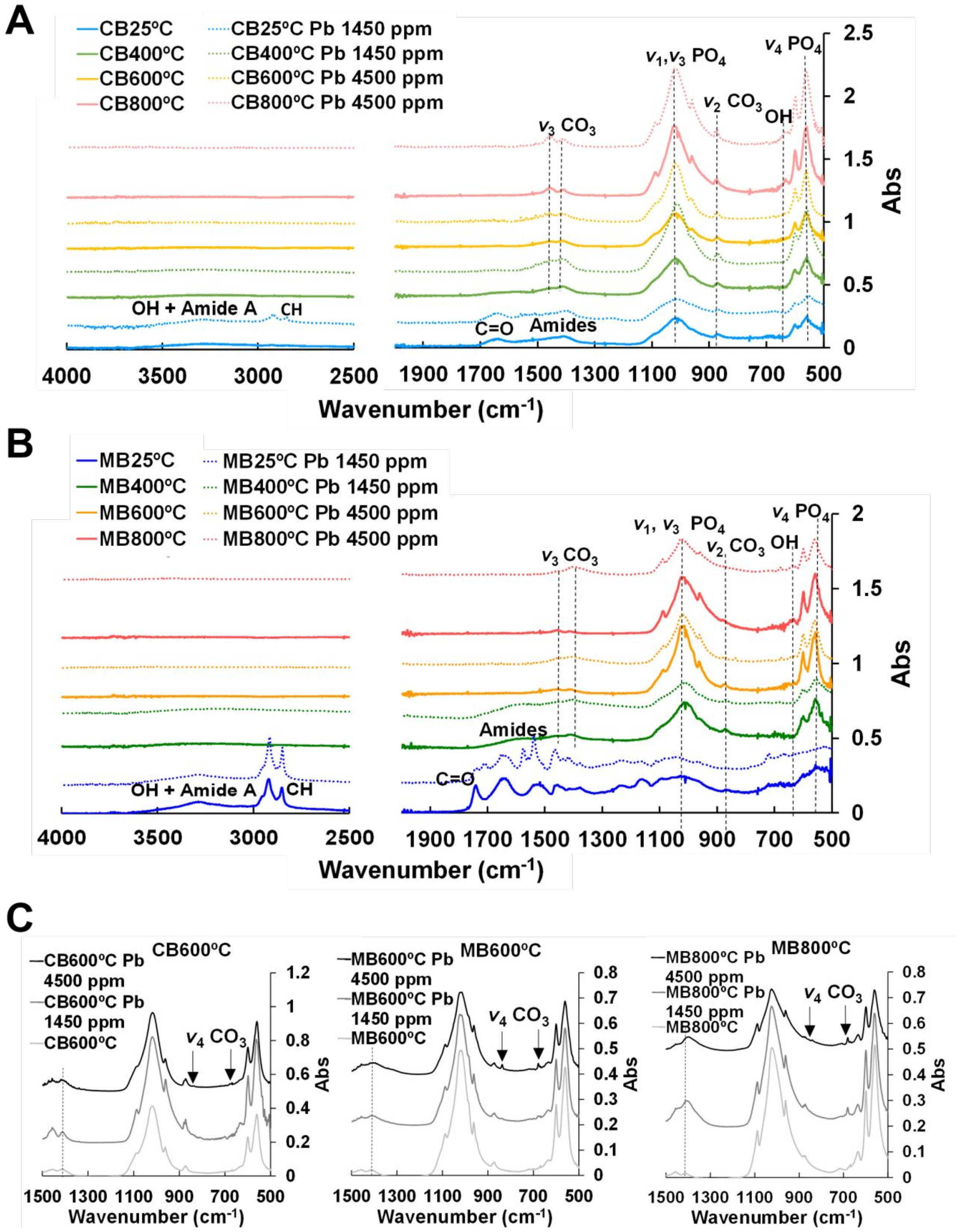

For Pb-free experiments, FT-IR analyses show the main IR adsorption bands produced by the different bone mineral and organic components. Regarding the latter, the intensity of these bands change depending on the temperature of the bone thermal

treatment. As the treatment temperature increases, the main bands at 3000-3500 cm^{-1} and 1200-1800 cm^{-1} decrease in intensity due to the combustion of the organic components (i.e., collagen and NCPs, lipids; [1]. There are also significant changes in the adsorption bands associated with the mineral part (phosphate and carbonate groups) due to the increasing crystallinity of bone mineral. For Pb-bearing experiments (particularly evident for MB600ºC and MB800ºC), new additional peaks appear around 837 cm^{-1} and 670-680 cm^{-1}, and a notable shift in the position of the main carbonate band from 1410 to 1390 cm^{-1} can be observed. This shift is due to the formation of Pb carbonate minerals such as hydrocerussite (1400-1350 $cm^{-1}$and 837 cm^{-1}), plumbonacrite (1400 cm^{-1} and 834 cm^{-1}) and cerussite (1400 cm^{-1} and 824 cm^{-1}). These minerals also exhibit a v_4 CO_3 peak at 678-690 cm^{-1} [9,10]. These results are consistent with XRD data showing the formation of these Pb mineral phases. However, these peaks were only observed in our experiments for samples exposed to solutions containing the highest Pb concentrations. Our findings are consistent with those of other authors [11], who also observed alterations in the intensity of the carbonate peaks at 1413 cm^{-1} and phosphate peaks at 1020 cm^{-1} due to the adsorption of Pb from solutions onto the bone mineral.

References

[1] A.B. Rodríguez Navarro, S. Madero, M. Greiner, P.A. Rodriguez-Jimenez, W.W. Schmahl, C. Jiménez-López, Effect of Heating on Avian (Cortical and Medullary) Bone Chemistry, Mineralogy and Structural Organization, Cryst. Growth Des. 23 (2023) 7841–7852.

[2] K. Kinashi, Y. Kambe, M. Misaki, Y. Koshiba, K. Ishida, Y. Ueda, Synthesis, characterization, photo-induced alignment, and surface orientation of poly(9,9-dioctylfluorene-alt-azobenzene)s, J. Polym. Sci. A Polym. Chem. 50 (2012) 5107–5114.

[3] Z.A. Alothman, A review: Fundamental aspects of silicate mesoporous materials, Mater. 5 (2012) 2874–2902.

[4] S. Mostofa, S.A. Jahan, B. Saha, N. Sharmin, S. Ahmed, Kinetic and thermodynamic investigation on adsorption of lead onto apatite extracted from mixed fish bone, Environ. Nanotechnol. Monit. Manag. 18 (2022) 100738.

[5] A. Doostmohammadi, A. Monshi, M.H. Fathi, O. Braissant, A comparative physico-chemical study of bioactive glass and bone-derived hydroxyapatite, Ceram. Int. 37 (2011) 1601–1607.

[6] N. Guzelsu, C. Wienstien, S.P. Kotha, A new streaming potential chamber for zeta potential measurements of particulates, Rev. Sci. Instrum. 81 (2010).

[7] R. Oftadeh, M. Perez-Viloria, J.C. Villa-Camacho, A. Vaziri, A. Nazarian, Biomechanics and Mechanobiology of Trabecular Bone: A Review, J. Biomech. Eng. 137 (2015) 010802.

[8] D.A. Zairin, S.W. Phang, Calcination time and temperature effect on natural hydroxyapatite obtained from fish bones for bone tissue engineering, J. Eng. Sci. Technol. 39 (2018) 51.

[9] M.H. Brooker, S. Sunder, P. Taylor, V.J. Lopata, Infrared and Raman spectra and X-ray diffraction studies of solid lead(II) carbonates, Can. J. Chem. 61 (1983) 494–502.

[10] O. Siidra, D. Nekrasova, W. Depmeier, N. Chukanov, A. Zaitsev, R. Turner, Hydrocerussite-related minerals and materials: structural principles, chemical variations and infrared spectroscopy, Acta Crystallogr. Sect. B Struct. Sci. Cryst.

Eng. Mater. 74 (2018) 182–195.
[11] B. Kizilkaya, A.A. Tekinay, Utilization to remove Pb (II) Ions from aqueous environments using waste fish bones by ion exchange, J. Chem. 2014 (2014).
